# Modulation of tumor microenvironment by targeting histone acetylation in bladder cancer

**DOI:** 10.1038/s41420-023-01786-3

**Published:** 2024-01-04

**Authors:** Sandra P. Nunes, Lucia Morales, Carolina Rubio, Ester Munera-Maravilla, Iris Lodewijk, Cristian Suárez-Cabrera, Victor G. Martínez, Mercedes Pérez-Escavy, Miriam Pérez-Crespo, Miguel Alonso Sánchez, Esther Montesinos, Edurne San José-Enériz, Xabier Agirre, Felipe Prósper, Antonio Pineda-Lucena, Rui Henrique, Marta Dueñas, Margareta P. Correia, Carmen Jerónimo, Jesús M. Paramio

**Affiliations:** 1 Cancer Biology and Epigenetics Group, Research Center of IPO Porto (CI-IPOP)/CI-IPOP@RISE (Health Research Network), Portuguese Oncology Institute of Porto (IPO-Porto)/Porto Comprehensive Cancer Center Raquel Seruca (Porto.CCC), Porto, Portugal; 2 Molecular Oncology Unit, Centro de Investigaciones Energéticas, Medioambientales y Tecnológicas (CIEMAT), Madrid, Spain; 3 Biomedical Research Institute I+12, University Hospital “12 de Octubre”, Madrid, Spain; 4 Doctoral Program in Biomedical Sciences, ICBAS - School of Medicine and Biomedical Sciences, University of Porto, Porto, Portugal; 5 Centro de Investigación Biomédica en Red de Cáncer (CIBERONC), Madrid, Spain; 6 Hemato-Oncology Program, Center for Applied Medical Research (CIMA), Universidad de Navarra, IDISNA, Pamplona, Spain; 7 Departmento de Hematología, Clínica Universidad de Navarra, and CCUN, Universidad de Navarra, Pamplona, Spain; 8 Small-Molecule Discovery Platform, Molecular Therapeutics Program, Center for Applied Medical Research (CIMA), Universidad de Navarra, Pamplona, Spain; 9 Department of Pathology, Portuguese Oncology Institute of Porto, Porto, Portugal; 10 Department of Pathology and Molecular Immunology, ICBAS - School of Medicine & Biomedical Sciences, University of Porto, Porto, Portugal

**Keywords:** Cancer microenvironment, Tumour immunology, Bladder cancer, Tumour immunology, Epigenetics

## Abstract

Alterations in the epigenetic machinery in both tumor and immune cells contribute to bladder cancer (BC) development, constituting a promising target as an alternative therapeutic option. Here, we have explored the effects of a novel histone deacetylase (HDAC) inhibitor CM-1758, alone or in combination with immune checkpoint inhibitors (ICI) in BC. We determined the antitumor effects of CM-1758 in various BC cell lines together with the induction of broad transcriptional changes, with focus on the epigenetic regulation of PD-L1. Using an immunocompetent syngeneic mouse model of metastatic BC, we studied the effects of CM-1758 alone or in combination with anti-PD-L1 not only on tumor cells, but also in the tumor microenvironment. In vitro, we found that CM-1758 has cytotoxic and cytostatic effects either by inducing apoptosis or cell cycle arrest in BC cells at low micromolar levels. PD-L1 is epigenetically regulated by histone acetylation marks and is induced after treatment with CM-1758. We also observed that treatment with CM-1758 led to an important delay in tumor growth and a higher CD8 + T cell tumor infiltration. Moreover, anti-PD-L1 alone or in combination with CM-1758 reprogramed macrophage differentiation towards a M1-like polarization state and increased of pro-inflammatory cytokines systemically, yielding potential further antitumor effects. Our results suggest the possibility of combining HDAC inhibitors with immunotherapies for the management of advanced metastatic BC.

## Introduction

Bladder cancer (BC) is the tenth most common cancer diagnosed worldwide, listing as the sixth most incident cancer in men [1]. About 75% of the patients are diagnosed with nonmuscle-invasive BC (NMIBC), whereas the remaining 25% display muscle-invasive BC (MIBC) [2]. Although NMIBC patients are associated with a good outcome, up to 70% of patients recur, with a significant percentage progressing to MIBC [3]. The treatment of MIBC comprises radical cystectomy and platin-based chemotherapy in a neoadjuvant or adjuvant setting [4]. Nevertheless, MIBC patients considered to be unfit for chemotherapy or surgery, presenting comorbidities or metastatic disease have few therapeutic options available. The implementation of immune checkpoint inhibitors (ICI), such as anti-programmed death-ligand 1 (PD-L1), as an option for patients positive for PD-L1 expression led to an increase of overall survival [5]. However, the percentage of patients with significant clinical response ranges between 17 and 26%, which is quite limited [6]. Thus, understanding the mechanisms behind BC development can unravel new therapeutic targets and strategies, with the main goal of improving patients’ survival.

Epigenetic landscape reprograming is one of the main mechanisms contributing to BC progression and is considered an emergent hallmark of cancer [7]. Histone acetylation is of the most prominent mechanisms involved in gene expression regulation favoring tumorigenesis [8]. Accordingly, histone acetylation is involved in transcriptional activation, through the addition of acetyl groups in lysine residues of histone tails by histone acetyltransferases (HATs). Histone deacetylases (HDACs), by removing acetylation marks, lead to transcriptional repression by turning DNA into a closed-chromatin state [9].

Since epigenetic marks represent an enticing target for cancer therapeutics, a group of drugs has been developed throughout the years targeting several epigenetic enzymes, called epigenetic modulating drugs or epidrugs [10]. The most well-known HDAC inhibitors (HDACi) comprise hydroxamic acids, including belinostat, vorinostat and panobinostat, all pan-HDACi FDA-approved for several haematological cancers [11]. HDACi also display antitumoral effects in BC cells [12, 13].

Most studies comprising epidrugs focus on the effects on cancer cells, yet the tumor microenvironment cells can also be affected since they are also epigenetically regulated. HDACi have been previously shown to modulate tumor microenvironment and improve response to ICI in BC and other cancer types [14–16]. Specifically, the combination of entinostat, a selective HDAC1/3 inhibitor, and anti-PD-1 therapy led to complete responses in an immunocompetent mouse model of BC [16]. Having into account the pre-clinical evidence, several clinical trials are being carried out to evaluate the efficacy of the combination of epidrugs and ICI in various solid tumors including BC [17]. Nevertheless, HDACi were shown to have low bioavailability, limited half-life and dose-limiting toxicity, hampering their use in clinical practice for cancer treatment [18]. Thus, the search for new epidrugs with more specific epigenetic targets at low therapeutic doses would fast-track their usage in clinical practice.

Here, we describe the antitumor efficacy of CM-1758, a novel HDAC inhibitor, in BC in vitro and in vivo models. First, the cytotoxic/cytostatic effects of CM-1758 were evaluated in several BC cell lines. Then, the effects on immune-related pathways were also studied, namely by assessing PD-L1 expression and its epigenetic regulation. Since CM-1758 up-regulated PD-L1, we postulated that combining CM-1758 and PD-L1 blockage could be a promising strategy for BC treatment. For that, an immunocompetent syngeneic mouse model of aggressive BC was used to assess CM-1758 effects in vivo alone or in combination with anti-PD-L1. The effects of CM-1758 were evaluated not only in tumor cells, but also in the tumor microenvironment comprising immune and non-immune cells and cytokine profile. CM-1758 displayed significant antitumor effects in vitro and in vivo concomitantly with tumor microenvironment remodeling.

## Results

### CM-1758 displayed cytotoxic/cytostatic effects in bladder cancer cells

**Fig. 1:**
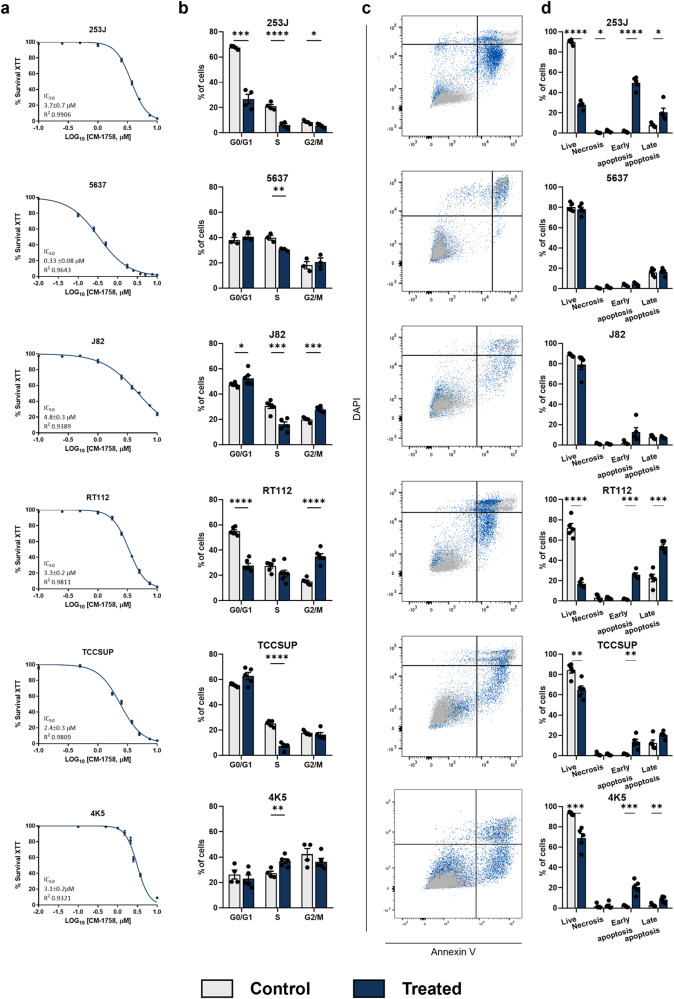
Cytotoxic and cytostatic effects of CM-1758 in bladder cancer cell lines. **a** Survival curves with the IC_50_ value for the BC cell lines 253 J, 5637, J82, RT112, TCCSUP and 4K5 (percentage of survival with calculated with XTT vs log_10_ of CM-1758 concentrations) with respective standard deviation and R^2^. **b** Cell cycle profiles containing G0/G1, S and G2/M phases for 253 J, 5637, J82, RT112, TCCSUP and 4K5 after treatment with CM-1758. **c** Representative gating of live (DAPI negative, annexin V negative), necrotic (DAPI positive, annexin V negative), early apoptotic (DAPI negative, annexin V positive) and late apoptotic cells (DAPI positive, annexin V positive) cells. **d** Percentage of live, necrotic, early and late apoptotic cells for all cell lines after treatment with CM-1758. Non-treated cells are represented in grey and CM-1758 treated cells are shown in blue. Treatment with CM-1758 was given for 48 h with IC_50_ dose calculated for each cell line for cell cycle and annexin V analyzes. Data shown are the mean of ≥3 experiments ± SEM. *P*-values are represented as ns – not significant, *<0.05, **<0.01, ***<0.001 and ****<0.0001.

The effects of the CM-1758 inhibitor were evaluated in a wide array of BC cell lines. CM-1758 demonstrated effects on the low micromolar range, with IC_50_ ranging between 0.33 and 4.8 µM (Fig. 1a; Supplementary Table S4). Remarkably, CM-1758 also affected the cell cycle, with a decrease in the percentage of S-phase cells observed for 253 J, 5637, J82 and TCCSUP cell lines after treatment (Fig. 1b). J82 cells also displayed higher percentage of G2/M phase in the treated cells, which was more evident for RT112 cells (Fig. 1b). The mouse 4K5 cell line disclosed cell arrest at the S-phase (Fig. 1b). Interestingly, 253 J and RT112 cells displayed a reduced percentage of G0/G1 phase upon treatment (Fig. 1b).

A decrease in the percentage of live cells in 253 J, RT112, TCCSUP and 4K5 treated cells was observed, in parallel with an increase in the percentage of cells in late apoptosis (Fig. 1c and d), with a more pronounced effect in 253 J and RT112 cell lines (Fig. 1c and d). 253 J, RT112, TCCSUP and 4K5 cells showed a higher percentage of early apoptosis, while only 253 J displayed higher levels of necrotic cells, with no apparent differences for the other cell lines (Fig. 1c and d). Altogether, these data demonstrate that CM-1758 has both cytotoxic and cytostatic effects in BC cell lines, either by inducing cell cycle arrest and/or apoptosis at low IC_50_ micromolar concentration.

### CM-1758 induced changes in the transcriptomic profile of bladder cancer cells

**Fig. 2:**
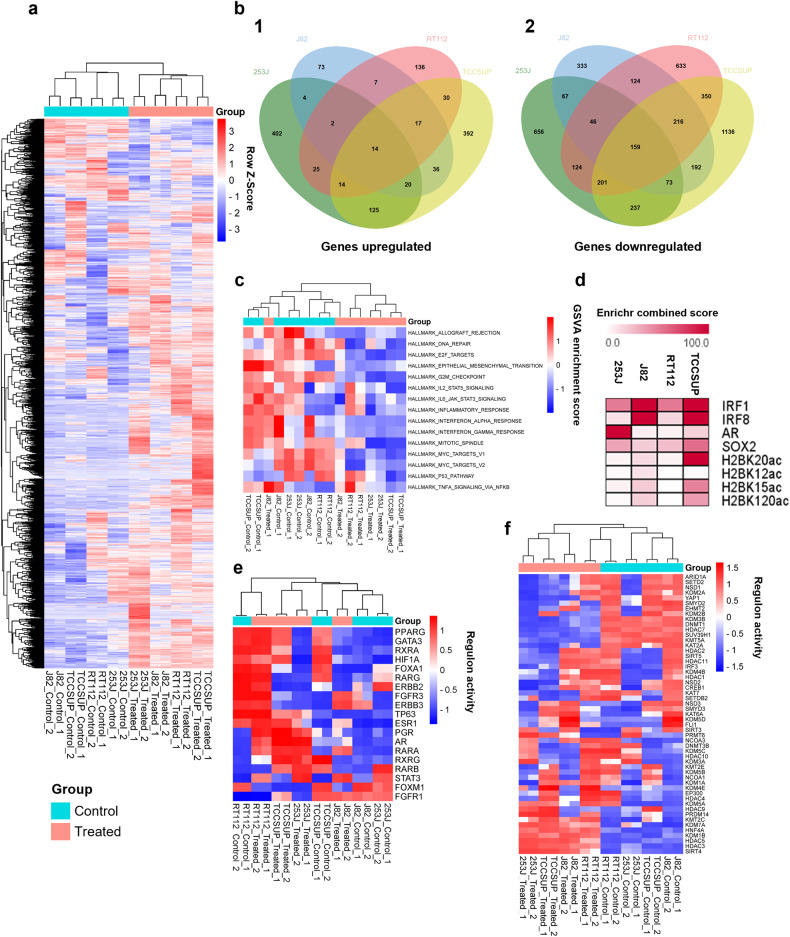
CM-1758 effects on the transcriptomic profile of 253 J, J82, RT112 and TCCSUP cells. **a** Heatmap of the dysregulated genes between untreated control vs. treated cells with IC_50_ dose of CM-1758. Genes and groups (control and treated) are hierarchically clustered. **b** Venn diagrams of genes (1) up and (2) downregulated in treated cells *vs*. control cells. **c** Heatmap of GSVA analysis showing the main signaling pathways from hallmarks in cancer enriched in control and treated cells. Control and treated groups are hierarchically clustered. **d** Enrichr analysis of the upregulated genes in all cell lines when compared to the respective untreated control cells. **e** Regulon activity profiles for potential regulators associated with BC. Control and treated groups are hierarchically clustered. **f** Regulon activity profiles for potential regulators associated with chromatin remodeling. Control and treated groups are hierarchically clustered. Control denotes untreated cells, whereas treated refers to CM-1758 treated cells. Treatment with CM-1758 was given for 48 h with IC_50_ dose calculated for each cell line. Two independent experiments were included for each cell line and condition.

Hereafter, we evaluated the transcriptomic profile of BC cells by RNA-seq after treatment with the IC_50_ doses of CM-1758. Treatment with CM-1758 drove marked changes in the transcriptomic profile of BC cells (Fig. 2a). Remarkably, there was limited overlap in the genes upregulated (Fig. 2b1) or downregulated (Fig. 2b2) considering all cell lines, although all cell lines showed homogeneous clustering in PCA analysis when comparing control vs. treated (Supplementary Fig. S5a–e). GSEA (Supplementary Fig. S6a–d) and GSVA analysis (Fig. 2c) revealed that the treatment decreased the expression of multiple genes involved in cell proliferation, such as E2F and MYC targets and epithelial-mesenchymal transition. Moreover, in some cell lines there was an effect on various immunological pathways such as IL-2/STAT5, IFN-α, IFN-γ and TNF- α signaling via NF- κB (Fig. 2c). However, these effects on immunological pathways were cell line dependent. Having into account the genes upregulated by CM-1758, further analyzes of the transcription factors and histone using the Enrichr webtool revealed a prominent role of IRF1 and IRF8 as well as acetylation of various histone H2B residues, more noticeable in TCCSUP cells (Fig. 2d). Also, analyzes of transcriptional regulatory networks (regulons) showed that some key elements in BC such as *FOXM1* were downregulated, while others, such as *STAT3*, were predominantly upregulated (Fig. 2e, Supplementary Fig. 6e). We also observed that many regulons modulating chromatin remodeling were also affected by treatment, including epigenetic machinery related not only to acetylation, but also histone and DNA methylation including lysine demethylases (KDMs) and DNA methyltransferases (DNMTs) (Fig. 2f). Overall, CM-1758 led to profound changes in the transcriptomic profile of BC cells, affecting pathways related to tumor cell aggressiveness and epigenetic machinery.

### CM-1758 treatment regulated PD-L1 expression by induction of histone acetylation

**Fig. 3:**
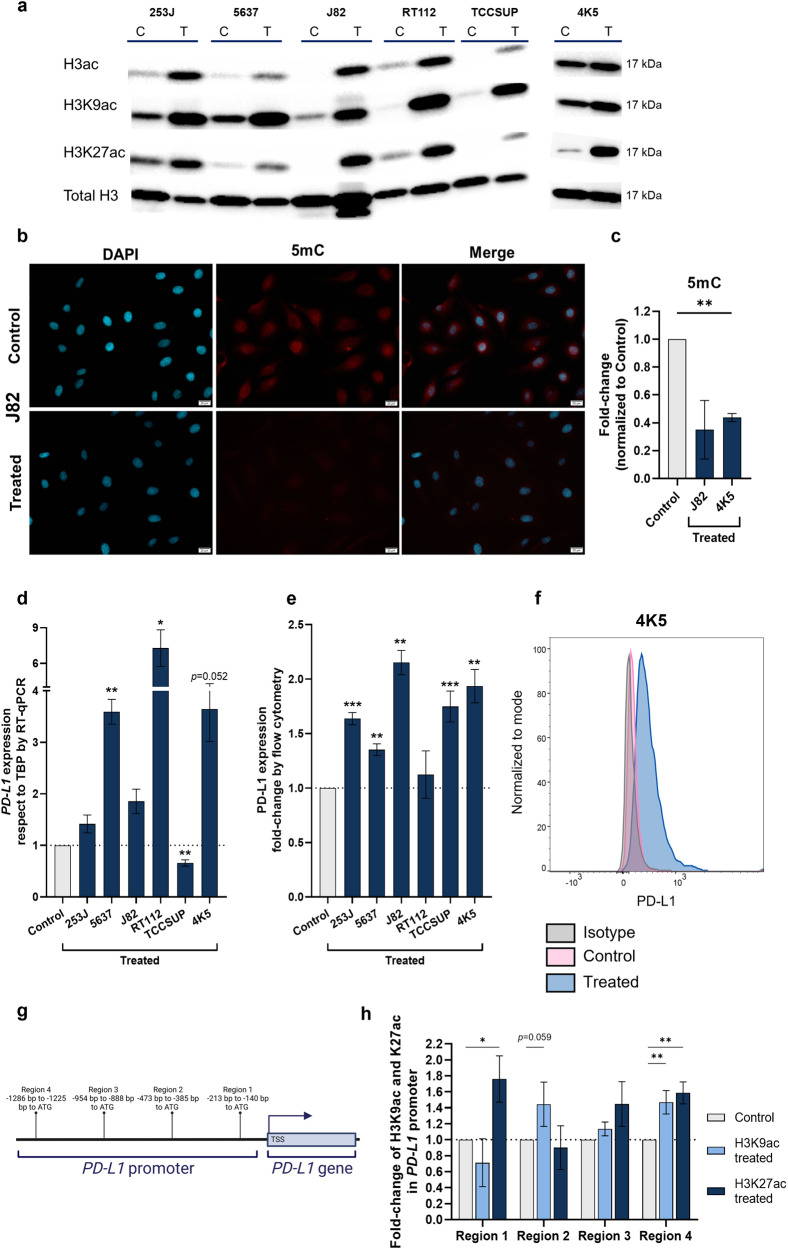
Histone acetylation and DNA methylation levels after treatment with CM-1758. PD-L1 epigenetic regulation by promoter acetylation. **a** Western blot analysis of total acetylation of H3 (H3ac), acetylation of lysine 9 of histone 3 (H3K9ac) and acetylation of lysine 27 of histone 3 (H3K27ac) respect to total histone 3 (H3) levels for all cell lines after treatment with CM-1758. C – Control, T - Treated. **b** Representative immunofluorescence for 5-methylcytosine of J82 cells after treatment with CM-1758 (in red). Cell nuclei were identified by DAPI staining (in blue). Scale bar 20 µm. **c** Quantification of 5-methylcytosine levels by immunofluorescence after treatment with CM-1758 represented by fold-change respective to the untreated control cells in J82 and 4K5 cells. **d** Relative expression by RT-qPCR of *PD-L1* respect to TBP for all BC lines after treatment with CM-1758. **e** PD-L1 expression evaluated by flow cytometry represented by fold-change respective to non-treated control cells and (**f**) Single parameter histogram of mean intensity fluorescence (MFI) of PD-L1 levels with CM-1758 treatment in 4K5 cells (**g**) Schematic representation of the *PD-L1* promoter regions evaluated by CUT&RUN (**h**) Presence of H3K9 and H3K27 acetylation in four different regions [region 1 -213 to -140 bp, region 2 -473 to -385 bp, region 3 -954 to -888 bp and region 4 -1286 to -1225 bp before the transcription start site (TSS)] of *PD-L1* promoter after treatment with CM-1758 evaluated by CUT&RUN. Values are represented as fold-change relative to the untreated control cells. Cells were treated with CM-1758 for 48 h with the IC_50_ dose calculated for each cell line. Data shown are the mean of ≥3 experiments ± SEM. *P*-values are represented as ns – not significant, *<0.05, **<0.01 and ***<0.001.

Next, we studied the effects of CM-1758 on histone acetylation in BC cell lines. We observed increased H3, H3K9 and H3K27 acetylation levels by western blot (Fig. 3a and Supplementary Fig. S7a) in all the analyzed cell lines after treatment with CM-1758. Of note, the expression of HDACs was altered in BC cells either by transcript or protein levels (Supplementary Fig. S7b and c). Since DNA methylation and histone modifications are two epigenetic mechanisms intertwined in the regulation of gene expression, we also explored the global levels of 5-methylcytosine in J82 and 4K5 BC cells. Immunofluorescence analyzes demonstrated that total 5-methylcytosine levels were decreased in J82 and 4K5 BC cells after CM-1758 treatment (Fig. 3b and c), which was paralleled by decreased DNMTs’ levels (Supplementary Fig. S7c and d). The lower levels of DNA methylation could be a direct consequence of the higher levels of acetylation observed after treatment with CM-1758. These results show that CM-1758 treatment affects histone acetylation, but also other epigenetic mechanisms, such as DNA methylation indirectly.

Some epidrugs were shown by others to have synergistic effects with immunotherapies, since drug treatments can induce immune checkpoints’ expression, conferring higher sensitivity to ICI [15, 16]. Furthermore, transcriptomic data showed that CM-1758 has effects on immune pathways in cancer cells. Thus, we investigated whether the global changes in acetylation and DNA methylation levels induced by CM-1758 treatment could have an impact on PD-L1 expression in BC cell lines, as previously reported for other solid tumors [19, 20]. Remarkably, we observed that PD-L1 levels increase in cells after CM-1758 treatment at both mRNA (Fig. 3d) and membrane protein (Fig. 3e and f) levels. Afterwards, we hypothesized whether this increased expression was attributable to epigenetic regulation of PD-L1. CUT&RUN experiments showed that H3K9ac and H3K27ac marks were increased at the PD-L1 promoter in 4 different regions, from 100 to 1200 bp upstream of initiation transcription site, after CM-1758 treatment (Fig. 3g and h). On the other hand, DNA methylation levels in these regions, assessed by qMSP, were not affected by treatment showing low levels of DNA methylation on the analyzed CGs (Supplementary Fig. S8a and b). These results indicated that CM-1758 increased PD-L1 levels through its effect on HDACs and, consequently, PD-L1 promoter acetylation marks.

### CM-1758 and anti-PD-L1 as a therapeutic strategy in a bladder cancer immunocompetent mouse model

**Fig. 4:**
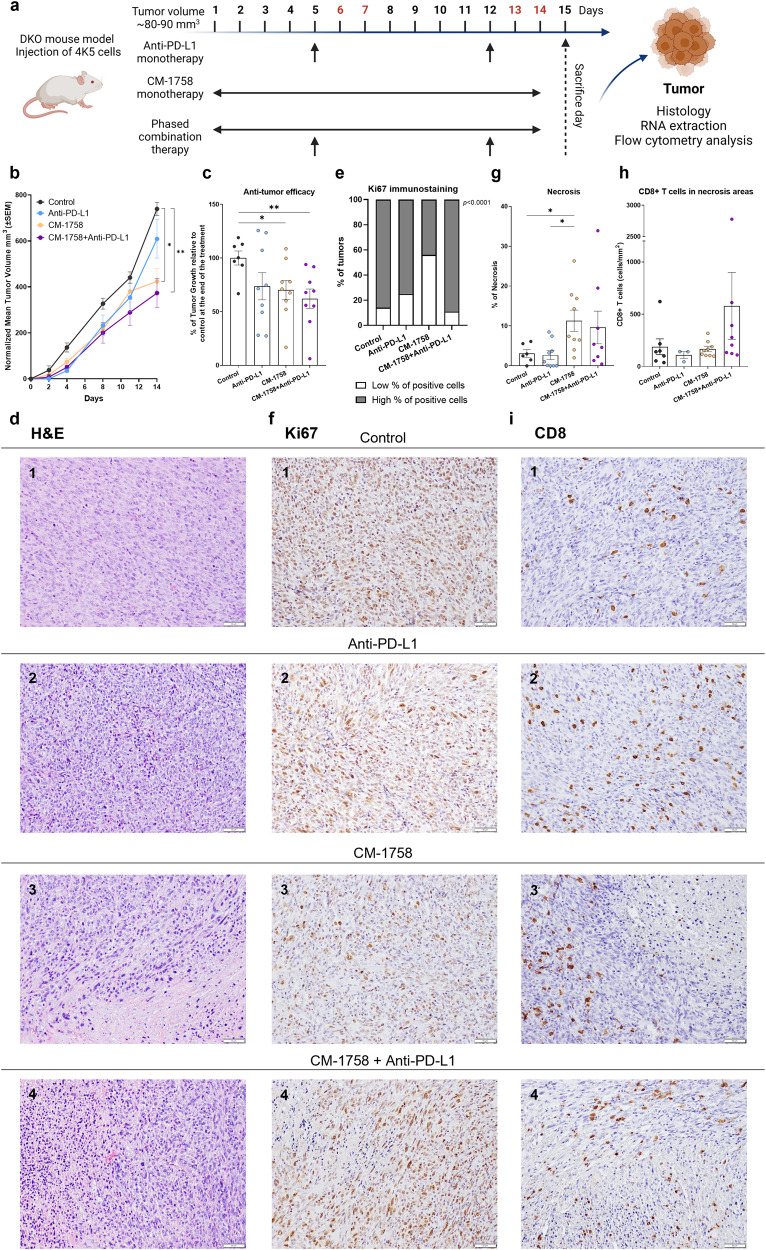
In vivo effects of CM-1758 alone and in combination with immune checkpoint inhibitors. **a** Schematic representation of the protocols used for treatment with CM-1758 and anti-PD-L1 alone or in combination. CM-1758 was applied for two weeks, five days a week with two rest days represented in red. Anti-PD-L1 was injected once a week for two weeks. Mice in all groups were sacrificed at day 15. Tumors were collected for histology, flow cytometry analysis and RNA extraction. Created with BioRender.com (**b**) Normalized tumor growth curves for the first day of treatment in control, anti-PD-L1, CM-1758 and CM-1758+anti-PD-L1 groups. **c** Anti-tumor efficacy of anti-PD-L1, CM-1758 and CM-1758+anti-PD-L1. **d** Representative H&E staining of the tumors from each group (1) control, (2) anti-PD-L1, (3) CM-1758 and (4) CM-1758+anti-PD-L1); **e** Graphical representation of percentage of Ki67 expression for each group. Cut-off was defined as <80% as low and >80% as high percentage of positive cells. **f** Representative immunohistochemistry staining of Ki67 for (1) control, (2) anti-PD-L1, (3) CM-1758, and (4) CM-1758+anti-PD-L1 tumors. **g** Percentage of necrosis present in tumors from each treatment group at the end of treatment of control, anti-PD-L1, CM-1758 or combination. **h** Number of CD8 + T cells (cells/mm^2^) infiltrating in necrosis areas in control, anti-PD-L1, CM-1758 and CM-1758+anti-PD-L1 groups. Only tumors displaying necrosis areas were evaluated. **i** Representative immunohistochemistry staining of CD8 for (1) control, (2) anti-PD-L1, (3) CM-1758, and (4) CM-1758+anti-PD-L1 tumors. Graphs show individual values as the mean ± SEM for ≥8 mice included in each group. Scale bar 50 µM. *P*-values are represented as ns – not significant, *<0.05, **<0.01 and ***<0.001.

The data obtained concerning increased PD-L1 expression together with gene expression changes as a consequence of CM-1758 treatment in vitro prompted us to study in vivo the effects of CM-1758 combined with anti-PD-L1 using a double knock-out (*Pten*^*F/F*^, *Trp53*^*F/F*^; DKO) immunocompetent BC mouse model. For that, GFP-expressing 4K5 cells (derived from a BC tumor developed in a DKO mouse injected with adenoviruses expressing Cre-recombinase under the control of keratin 5 promoter) were subcutaneously injected in the flank of syngeneic mice and treated with CM-1758 and/or PD-L1 blockade (Fig. 4a). Even with only two weeks of treatment period, an anti-tumoral effect of CM-1758 was observed, which was slightly increased by the combination with anti-PD-L1 (Table 1, Fig. 4b, c and d1, 2, 3 and 4; Supplementary Fig. S9a, b, c, and d). Both Ki67 and BrdU immunohistochemistry analyses demonstrated a significant decrease in tumor cell proliferation levels in CM-1758 treatment group (Fig. 4e and f1, 2, 3 and 4; Supplementary Figs. S9e and S9f1, 2, 3 and 4). Furthermore, more prominent immune infiltrates were found in the tumors treated with the combination, confirmed by the immunostaining analysis for CD8 + T cells (Supplementary Fig. 9g). The assessment of necrotic areas revealed that CM-1758, either alone or in combination with anti-PD-L1, resulted in a higher percentage of necrotic regions within the examined tumors (Fig. 4g). Interestingly, the combination-treated tumors displayed a clear tendency revealing a higher infiltration of CD8 + T cells in necrotic areas compared to control, anti-PD-L1, and CM-1758-treated tumors (Figs. 4h and i1, 2, 3 and 4). However, it did not reach statistical significance, and there was no observed correlation between these variables (Fig. 4h; Supplementary Fig. 9h, i, j, and k).

**Table 1:** Antitumor efficacy of anti-PD-L1, CM-1758 and CM-1758+anti-PD-L1 in comparison with the control group.

Treatment group	Mean % T/C ( ± SD)	*p* value		
		vs. Control	vs. Anti-PD-L1	vs. CM-1758
Anti-PD-L1	74 ± 38	0.112	-	-
CM-1758	70 ± 27	0.023	0.822	-
CM-1758+Anti-PD-L1	62 ± 27	0.006	0.463	0.532

Percentage calculated as [T (tumor) / C (control)] x 100 of growth inhibition by anti-PD-L1, CM-1758 or the combination. Values represented as mean ± SD and *p*-values < 0.05 were considered statistically significant.

### CM-1758 treatment increased CD8+ cytotoxic T cells presence in the tumor microenvironment and promoted pro-inflammatory cytokine release

**Fig. 5:**
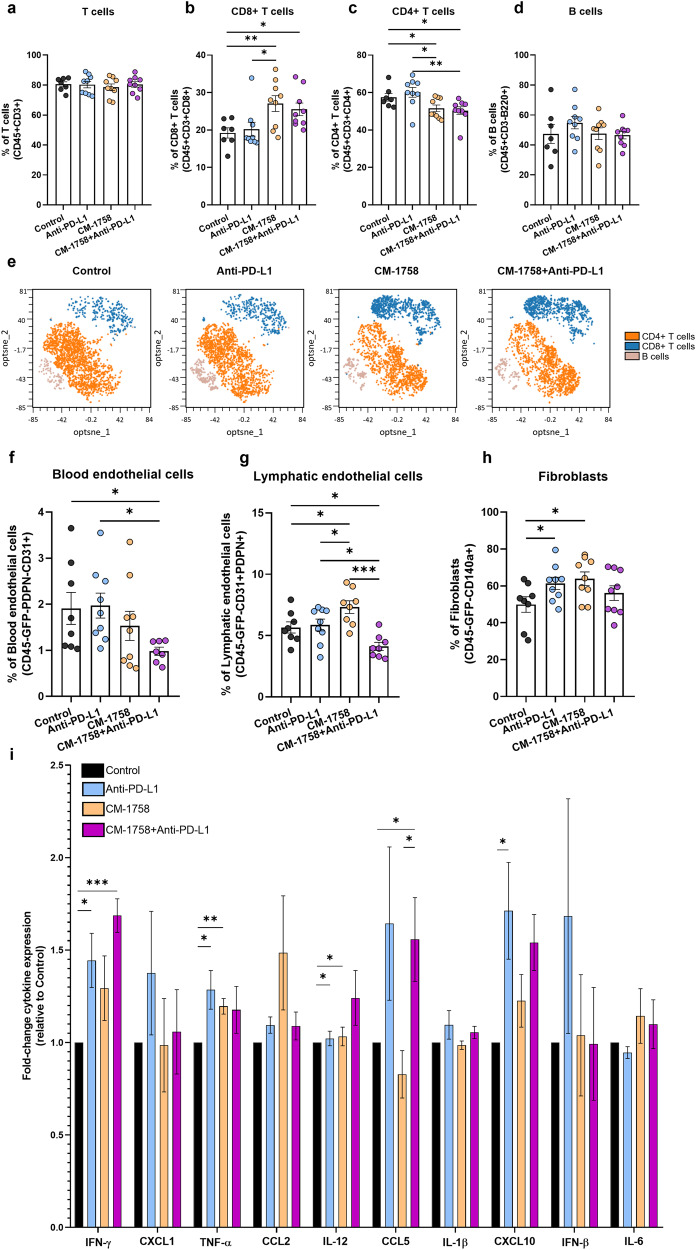
Effects of CM-1758 and immune checkpoint inhibitors in tumor lymphoid cells, the non-immune compartment and cytokine profile. Percentage of (a) T cells gated in CD45+ cells, (**b**) CD8 + T cells gated in CD3+ cells, (**c**) CD4 + T cells gated in CD3+ cells and (**d**) B cells gated in CD45+ cells present in the tumors. (**e**) Representative opt-tSNE analysis of CD8 + T, CD4 + T and B cells repertoire according to treatment groups. Percentage of (**f)** blood endothelial cells gated in CD45-GFP- cells, (**g**) lymphatic endothelial cells gated in CD45-GFP- cells (**h**) fibroblasts gated in CD45-GFP- cells present in the tumor microenvironment. (**i**) Fold-change of cytokine profile (IFN-γ, CXCL1, TNF-α, CCL2, IL-12, CCL5, IL-1β, CXCL10, IFN-β and IL-6) analyzed in plasma of control, anti-PD-L1, CM-1758 and CM-1758+anti-PD-L1 treated mice. Graphs show individual values as the mean ± SEM for ≥8 mice included in each group. *P* values are represented as ns – not significant, *<0.05, **<0.01 and ***<0.001.

Treatment with CM-1758, alone or in combination with anti-PD-L1, led to changes in tumor lymphoid repertoire. We did not find any differences in CD3 + T cells percentage respect to the CD45+ leucocytes population (Fig. 5a). However, when we looked for the type of lymphocytes present in the tumor, we did observe a reduction in CD4 + T cells, alongside with an increase in CD8 + T cells in tumors treated with CM-1758 or upon combination treatment (Fig. 5b and c), whereas no significant differences were observed for the B cell percentage (Fig. 5d). Thus, CM-1758 led to increased CD8+ cytotoxic T cells presence independently of immunotherapy, whereas CD4 + T cells detection was decreased (Fig. 5e).

The non-immune compartment of the tumor microenvironment also underwent changes upon ICI and CM-1758 treatments (Fig. 5f, g and h). The percentage of blood and lymphatic endothelial cells in the tumor significantly decreased after treatment with CM-1758+anti-PD-L1 in comparison with the control and anti-PD-L1 groups (Fig. 5f and g). On the other hand, the percentage of lymphatic endothelial cells was increased in the CM-1758 treated group in comparison with control, anti-PD-L1 and combination groups (Fig. 5g). The percentage of fibroblasts was increased in both anti-PD-L1 and CM-1758 groups (Fig. 5h). Almost all fibroblasts found in the tumor microenvironment were positive for α-SMA, a cancer-associated fibroblasts (CAFs) marker (Supplementary Fig. S10a; mean percentage of cells positive for α-SMA higher than 97%).

Next, we explored whether the treatment with CM-1758 might led to the release of pro-inflammatory cytokines systemically. Thus, multiple cytokines were measured in plasma upon different treatments using a cytometric bead-based assay (mean concentrations of each cytokine per treatment group are displayed in Supplementary Table S5). Anti-PD-L1 treatment led to an increase in IFN-γ, which was further boosted by the combination with CM-1758 (Fig. 5i). Moreover, increased TNF-α and IL-12 levels were observed in both anti-PD-L1 and CM-1758 treatment groups. The combination treatment increased CCL5 levels mainly by action of the anti-PD-L1 therapy and with no changes in CCL5 levels on the CM-1758 group, suggesting that CCL5 is regulated by ICI (Fig. 5i). The same seems to happen with CXCL10, which levels were significantly increased in the anti-PD-L1 treatment group, with the same tendency observed for the combination (Fig. 5i). These results highlight the importance of epigenetic mechanisms and ICI in cytokines’ expression regulation, which remains a poorly explored topic in BC.

### Anti-PD-L1 alone or in combination with CM-1758 reprogramed macrophages towards a M1-like polarization

**Fig. 6:**
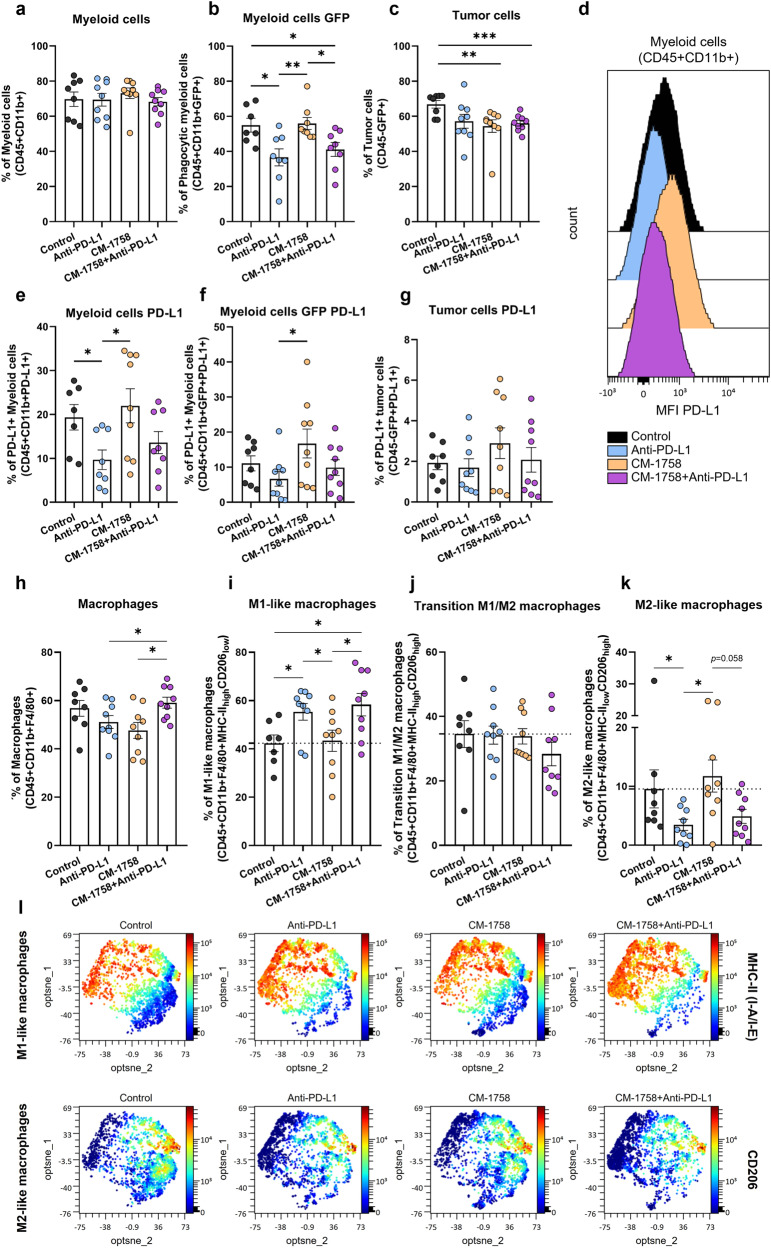
Myeloid repertoire changes after treatment with CM-1758 and PD-L1 blockade therapy. Percentage of (**a**) myeloid cells gated in CD45+ cells, (**b**) phagocytic myeloid cells gated in CD45+ cells and (**c**) tumor cells gated in live cells. (**d)** Single parameter histograms of mean intensity fluorescence (MFI) of PD-L1 expression in the four treatment groups. Percentage of PD-L1 positive of (**e**) myeloid cells gated in CD45 + CD11b+ cells, (**f**) phagocytic myeloid cells gated in CD45 + CD11b+GFP+ cells, and (**g**) tumor cells gated in GFP+ cells. Percentage of (**h**) macrophages gated in CD45 + CD11b+ cells, (**i**) M1-like macrophages gated in CD45 + CD11b + F4/80+ cells, **j** transition M1/M2 macrophages gated in CD45 + CD1b + F4/80+ cells and (**k**) M2-like macrophages gated in CD45 + CD11b + F4/80+ cells. **l** Opt-tSNE analyzes of tumor-present M1-like and M2-like macrophages for MHC-II (I-A/I-E) and CD206 markers. Graphs show individual values as the mean ± SEM for ≥8 mice included in each group. *P*-values are represented as ns – not significant, *<0.05, **<0.01 and ***<0.001.

We also investigated whether anti-PD-L1 and/or CM-1758 treatments could induce myeloid compartment remodeling. Although no differences were apparent in the percentage of myeloid cell present in the tumor (Fig. 6a), a lower percentage of phagocytic myeloid cells, identified by their positivity for GFP arising from the phagocytosis of GFP-expressing tumor cells, was found for anti-PD-L1 treated groups alone or in combination with CM-1758 (Fig. 6b). Furthermore, a significant decrease in the percentage of tumor cells was found for the treatment groups, including CM-1758 alone or in combination as compared with non-treated control mice (Fig. 6c).

Anti-PD-L1 treatment alone or in combination with CM-1758 led to significantly lower detectable PD-L1 levels in myeloid cells, both in all myeloid cells and phagocytic myeloid cells (Fig. 6d–f). No significant changes were found in tumor cells’ PD-L1 levels, showing that the anti-PD-L1 treatment mainly affected the myeloid cell population (Fig. 6g). Additionally, no changes at PD-L1 transcriptional levels were observed in the tumor bulk, indicating that the decreased cell surface PD-L1 detection might be due to anti-PD-L1 antibody still bound to the cells or to PD-L1 loss at the cell membrane by internalization processes (Supplementary Fig. S10b). On the other hand, CM-1758 alone seemed to slightly increase PD-L1 levels in tumor cells as observed in the in vitro conditions, although without statistical significance (Fig. 6g).

Compared with untreated samples, single treatment with anti-PD-L1 or CM-1758 led to a partial decrease in total macrophages (Fig. 6h). Interestingly, anti-PD-L1 (alone or in combination) treatment increased the presence of M1-like macrophages, while decreasing M2-like macrophages (Fig. 6i–l). Contrarily, CM-1758 alone did not seem to have a major impact in macrophages polarization (Fig. 6i–l). These findings suggest that the shift of macrophages toward an M1-like pro-inflammatory polarization is dependent on ICI treatment, with no observed syngeneic effect in combination with CM-1758.

## Discussion

BC represents a major public health concern due to its high incidence and prevalence, with current treatment options falling short to avoid disease recurrence and progression [1]. Although implementation of immunotherapy in advanced BC improved patients’ overall survival, a significant percentage of BC patients does not respond or become unresponsive within the course of ICI treatment [21]. Thus, new treatment strategies, such as epidrugs, alone or in combination with immunotherapy, are currently being explored [17]. Several HDACi are FDA-approved for treatment of hematologic malignancies, and are under clinical trials for BC patients, with a confirmed improvement of overall survival [22]. Furthermore, several in vivo and in vitro studies showed the synergistic effect of commercial HDACi and immunotherapy [14, 15]. Herein, we present encouraging data showing the anti-cancer effects of CM-1758, a novel inhibitor of HDACs, both in in vitro and in vivo models of advanced BC.

CM-1758 effects leading to cell cytotoxicity and cytostasis by either apoptosis or cell cycle arrest is in line with previous reports on HDACi such as vorinostat (SAHA) or entinostat [12, 13, 23–26]. Additionally, CM-1758 had inhibitory effects on HDACs and further transcriptomic analyzes revealed that not only the epigenetic machinery associated with acetylation was altered, but also the machinery responsible for the regulation of methylation of both histones and DNA. It is known that histone acetylation and DNA methylation are epigenetic mechanisms closely related in regulating gene expression, *i.e*., elevated histone acetylation can trigger DNA demethylation, promoting gene expression [27].

PD-L1 blocking antibodies are among the most frequently used ICI in BC [28] and, importantly, PD-L1 expression was shown to be regulated by epigenetic mechanisms [29, 30]. In line with previous studies using HDACi, we found increased PD-L1 expression levels upon CM-1758 treatment in several BC cell lines, consequence of higher acetylation levels in the PD-L1 promoter [30]. Histone acetylation increases gene expression since it neutralizes lysine residues positive charge, decreasing the interaction of histone proteins with DNA, making it more accessible for transcription factors [9, 31]. Conversely, although PD-L1 expression has been reported to be regulated by DNA methylation [29], no apparent effect was seen in PD-L1 promoter DNA methylation levels with CM-1758 treatment.

Since CM-1758 led to increased PD-L1 expression, the in vivo effects of a combinatory therapy of CM-1758 with anti-PD-L1 antibody were investigated. A subcutaneous syngeneic mouse model was used to evaluate the efficacy of CM-1758, with the main advantage of having a fully immunocompetent system and enabling easy following of tumor growth. Treatment with CM-1758 alone has significant anti-tumor effects, with better results than anti-PD-L1 blockade alone and a slightly synergistic effect when combined with ICI. Because a significant proportion of BC patients do not respond to ICI due to low PD-L1 expression levels or reduced immune cell infiltration (immune deserted and excluded tumors), our results suggest that CM-1758 may be a promising alternative approach to ICI.

It is acknowledged that epigenetics plays a major expression regulatory role not only in tumor cells, but also in tumor microenvironment cells including immune and nonimmune compartment [17]. In our hands, both CM-1758 and anti-PD-L1 induced changes in both immune and non-immune cells. Importantly, CM-1758 incremented CD8+ cytotoxic T cells infiltration, which is a rather favorable prognostic factor in BC [32]. Also, we found an increase in CD8 + T cell infiltration within the necrotic regions of tumors following the combined administration of CM-1758 and anti-PD-L1. Necrotic areas often exhibit immune suppression, either through the recruitment of M2 macrophages or due to the absence of active T cells [33], characteristics commonly associated with high-risk pathological features in upper tract urothelial carcinoma [34]. Our findings indicate that the combination treatment effectively counteracts the immune-suppressive effects associated with tumor necrosis, signifying a potential synergistic effect between CM-1758 and anti-PD-L1. In contrast, the decrease of CD4 + T cells after treatment may be associated with a reduction in regulatory T cells (T_reg_), as previously observed after in vivo entinostat treatment in a different BC model [16]. CM-1758 showed the ability to regulate the lymphoid repertoire, further supporting that epigenetic factors modulate BC tumors’ immune infiltrate features, specifically of T cells [35]. Henceforth, epidrugs to target tumor immune cells constitutes a strategy that should be exploited in conjunction with other immunotherapies in BC.

Myeloid cells are also key players for the effectiveness of immunotherapy [36]. A lower percentage of phagocytic myeloid cells was observed in the anti-PD-L1 treated groups, alone or in combination with CM-1758. Additionally, in our experimental conditions, phagocytic myeloid cells seemed to be the main population affected by anti-PD-L1 antibody treatment. This might be consequence of antibody-dependent cell-mediated cytotoxicity (ADCC) against the phagocytic myeloid cells. Indeed, avelumab was previously shown to mediate ADCC in tumor cells, which is in line with our findings [37]. Importantly, anti-PD-L1 alone or in combination with CM-1758 impacted macrophage status, shifting from an M2 anti-inflammatory to an M1 pro-inflammatory profile and sustaining a “hot tumor” paradigm, as already reported by other studies for ICI [15, 38]. Although epigenetic regulation has been previously implicated in macrophage polarization [15], in our hands CM-1758 did not alter macrophage differentiation. Moreover, despite observing a trend within the CM-1758 group, we did not find a significant change in the PD-L1 expression in tumor cells in vivo, which could be attributed to the fact that PD-L1 expression increase may be transient. Understanding when the PD-L1 levels detected are the highest by induction of CM-1758 could provide insights into alternative treatment regimens that may potentiate the synergy between CM-1758 and ICI. The non-immune compartment has a crucial role in tumor maintenance, either through angiogenesis, by providing nutrients and oxygen, or by supporting tumor cell growth [39]. CM-1758 altered the non-immune cell compartment by reducing the percentage of blood endothelial cells, which may decrease the tumor access to nutrients and diminishing metastasis development via blood vessels. Nonetheless, the percentage of lymphatic endothelial cells was increased in the CM-1758 treated group. The role of lymphatic endothelial cells remains under discussion since it increases the amount of tumor antigens in circulation, augmenting the probability of immune system activation [40], although it has been also implicated in tumor cell dissemination. Further studies are needed to explore the role and the impact of epigenetic regulation in lymphatic endothelial cells. Interestingly, we found that virtually all fibroblasts were positive for α-SMA, being classified as myofibroblast-like CAFs (myCAFs) [41]. MyCAFs play a crucial role in the tumor microenvironment by contributing to the deposition and organization of extracellular matrix components, including collagen and fibronectin, which provide structural support to the growing tumor mass in mouse subcutaneous tumor models [42]. In this line, the increase in the percentage of myCAFs in the treatment groups could be associated with a remodeling in the tumor’s extracellular matrix by treatments’ actions. However, comprehensive studies focusing on the effects of HDACi on CAFs in the context of BC in mouse orthotopic models are required to be better elucidate the contribution of these cells within the BC tumor microenvironment.

The cytokine profile was evaluated in plasma, indicating a systemic immune activation status of mice upon therapy by increasing pro-inflammatory cytokines in circulation. IFN‐γ and TNF-α are mainly produced by CD4 + T helper type 1 (Th1) cells, CD8+ cytotoxic T cells and NK cells, stimulating M1 macrophages’ antitumor effects and promoting the cytotoxic activity of CD8 + T cells and NK cells [43, 44]. Thus, the increase in cytokines levels is in accordance with increased CD8 + T cells and M1-macrophages depicted by the tumor microenvironment analysis. In our study, both CCL5 and CXCL10 levels seemed to be regulated by ICI. CCL5 is often seen as a “double-edge sword” in cancer, since it has been associated both with promoting antitumor immunity, as well as being implicated in tumor growth and migration [45]. In BC cell lines, conditioned media from TAMs increased invasiveness concomitantly with augmented CCL5 levels [46], although in BC patients high CCL5 expression in tumors associated with better prognosis [47]. In contrast, CXCL10 was shown to increase infiltration of CD8 + T cells, increasing migration and activation of immune cells in the tumor microenvironment [48, 49]. No relevant alterations in cytokines’ levels were exclusive to the combination treatment, suggesting that cytokines were independently regulated by ICI and/or CM-1758. Additional studies are required to unravel the exact role of cytokine epigenetic regulatory mechanisms in response to ICI and epidrugs in MIBC.

To conclude, our study describes a novel epigenetic inhibitor targeting HDACs, disclosing antitumor properties in BC both in vitro and in vivo models. Although CM-1758 was shown to have only a slight synergistic effect when combined with ICI, this epidrug may constitute an alternative to current ICI treatment schemes prescribed for BC patients. Besides having a direct impact in tumor growth, CM-1758 also leads to immune system activation. Undoubtedly, epigenetic mechanisms have an important role in regulating the tumor microenvironment and constitute an attractive target to modulate the immune system, with the final goal of tumor elimination and improving BC patients’ quality of life. Further investment in novel epidrugs, with extended bioavailability, reduced off-target effects and amenable to be tested in clinical trials as monotherapy, or in combination with chemotherapy and/or ICI, is mandatory to allow for the development of new and more effective strategies for BC management.

## Material and methods

### Cell culture

American type culture collection (ATCC) human BC cell lines 253 J, 5637, J82, RT112 and TCCSUP, including several tumor stages and BC subtypes [50] acquired in 2008, were selected for the purpose of this study. Cells lines were authenticated by short tandem repeat analysis (Unidad de Genómica, CIMA Lab Diagnostics) and routinely checked for the presence of mycoplasma using Venor®GeM One Step (Minerva Biolabs, Berlin, Germany). Additionally, a previously established mouse bladder tumor cell line 4K5 (*Pten* ^-/-^; *Trp53* ^-/-^; Adeno-Krt5-Cre) expressing GFP was also included in the study. 253 J, J82, RT112 and 4K5 cells were maintained in DMEM GlutaMAX™ medium (Thermo Fisher Scientific, Waltham, Massachusetts, USA), whereas TCCSUP cells were grown in EMEM medium (Lonza, Basel, Switzerland) and 5637 cells were maintained in RPMI 1640 medium (Thermo Fisher Scientific), all supplemented with 10% fetal bovine serum (FBS) and 1% of Antibiotic Antimycotic solution (Thermo Fisher Scientific), at 5% CO2 and 37°C in a humidifying chamber.

### Cell treatment with CM-1758 and IC_50_ calculation

CM-1758 (HDACs inhibitor) was stored in DMSO (Sigma Aldrich, Burlington, Massachusetts, USA) at the concentration of 100 mM at -20°C. The chemical structure was described previously [Compound 13e [18]]. The half-maximal inhibitory concentration (IC_50_) of CM-1758 was calculated for all cell lines. Cells were plated in 96-well plates at different densities according to cell line and, after 24 h, cells were treated with concentrations of CM-1758 ranging from 0.01 to 10 µM in complete medium, and left in culture for 48 h. Two controls were added per plate, including one with complete medium and another containing 0.01% of DMSO. Cell Proliferation Kit II XTT (Roche, Basel, Switzerland) was then used according to the manufacturer’s instructions. Absorbance was measured at 490 nm using a Genios Pro microplate reader (Tecan, Männedorf, Switzerland). At least four experimental replicates were used for IC_50_ calculation.

### Cell cycle analysis

All cell lines were seeded in 6-well plates and treated with CM-1758 for 48 h with the IC_50_ concentration calculated for each cell line, or with DMSO control. After that, cells were trypsinized (Trypsin-EDTA Solution, Sigma-Aldrich), collected and counted. Cells from control and treated conditions were collected for cell cycle analysis. Next, cells were washed with PBS and fixed with ethanol 70% for 1.5 h at 4°C. Cells were treated with RNAse and incubated at 37°C for 20 min with shaking. Finally, propidium iodide (Sigma Aldrich) at 50 µL/mL was added and incubated for 15 min in the dark. Cell cycle was analyzed in a LSR Fortessa cell analyzer (Becton Dickinson (BD) Biosciences, Franklin Lakes, New Jersey USA). Results were analyzed using FlowJo™ v10.8.1 (BD Biosciences). Five experimental replicates were used for data analysis.

### PD-L1 expression and Apoptosis assay by flow cytometry

Cells were seeded in 6-well plates and treated with CM-1758 at IC_50_ concentration for 48 h, or DMSO as a control, for evaluation of PD-L1 expression and apoptosis. Cells were trypsinized, collected and washed with PBS. Then, cells were incubated with 1 µL of human or mouse FcR blocking reagent (Miltenyi Biotec, Bergisch Gladbach, Germany) for 10 min at room temperature (RT). Samples were stained with human anti-PD-L1 (clone MIH3; Biolegend, San Diego, California, USA) or mouse anti-PD-L1 antibody (clone MIH5; BD Biosciences) for 30 min at 4°C in the dark. After washing, cells were incubated with a mix of Annexin Binding Buffer 1x (BD Biosciences), DAPI (Roche) and 2 µL of PE Annexin V (Ref 556421, BD Biosciences) for mouse, and FITC Annexin V (Ref 556419, BD Biosciences) for human cell lines, for 15 min in the dark at RT. Cells were acquired in a BD LSR Fortessa cell analyzer, and data analysis was performed using FlowJo^TM^ v10.8.1 (BD Biosciences). For apoptosis, live cells were identified as AnnexinV-DAPI-, early apoptotic as AnnexinV+DAPI-, late apoptotic as AnnexinV+DAPI+ and necrotic by Annexin-DAPI + . PD-L1 expression was analyzed only in live cells (DAPI-). Single stained and fluorescence minus one (FMO) controls were included.

### RNA extraction and RNA-sequencing sample preparation

Total RNA was extracted using the miRNAeasy Mini kit (Qiagen, Hilden, Germany), according to the manufacturer’s instructions. RNA concentration was measured on a Qubit 4 Fluorometer (Thermo Fisher Scientific), using a Qubit RNA broad range assay kit (Thermo Fisher Scientific) and stored at -80°C. RNA integrity number (RIN) was measured using the Qubit RNA IQ assay kit (Thermo Fisher Scientific) and samples with values higher than 8 were considered for RNA sequencing (RNAseq) analysis. One µg of total RNA was sent for RNAseq analysis by Macrogen RNA-seq services (Seoul, South Korea) using the Truseq stranded total RNA Library with Ribo-Zero Gold (Illumina, San Diego, California, USA) and the NovaSeq 6000 (Illumina) of two independent biological replicates for each condition (control and treated with CM-1758) of 253 J, J82, RT112 and TCCSUP.

### Real-time quantitative PCR (RT-qPCR)

One μg of RNA was used to synthetize cDNA using the High Capacity cDNA Reverse Transcription kit (Applied Biosystems™, Waltham, Massachusetts, USA) according to the manufacturer’s recommendations. Four µL of diluted 20x cDNA, 5 µL of GoTaq PCR Master Mix (Promega, Madison, Wisconsin, USA) and primers (Supplementary Table S1) at 0.5 µM were used per well. The RT-qPCR reactions were run in a QuantStudio™ 6 Flex Real-Time PCR (Thermo Fisher Scientific), along with the melting curves. *TBP* for mouse and human was used as housekeeping gene to normalize the results, and the relative expression for each transcript was calculated using the 2^–∆∆Ct^, as previously described [51]. Primers sequences can be found in Supplementary Table S1.

### CUT&RUN

Cleavage under targets & release using nuclease (CUT&RUN) was performed using the CUT&RUN assay kit (Cell Signaling Technology, Danvers, Massachusetts, USA), according to the manufacturer’s instructions. Briefly, 100 000 of J82 cells treated with the IC_50_ dose of CM-1758, or DMSO 0.01% as control, were collected, washed and incubated with 10 µL of concavalin A beads. Then, bead-bound cells were incubated with the primary antibodies for H3K9ac (dilution 1:50, #9649, Cell Signaling Technology), H3K27ac (dilution 1:100, #8173, Cell Signaling Technology), H3K4me3 for positive control (dilution 1:50, #9751, Cell Signaling Technology), or Rabbit IgG Isotype Control (dilution 1:50, #66362, Cell Signaling Technology), overnight at 4°C with rotation. After a wash, the cell-bead mixture was incubated with pAG-MNase at 4°C for 1 h. Next, after washing with wash buffer, calcium chloride was added, and samples were incubated at 4°C. After 30 min, stop buffer was added and DNA was purified using the QIAquick PCR Purification kit (Qiagen). For input samples preparation, cells were incubated with DNA extraction buffer (plus proteinase K and RNAse A) for 1 h at 55°C. Then, for cell lysis, cells were sonicated, and DNA was purified. Two µL of purified DNA, 5 µL of GoTaq PCR Master Mix (Promega) and 2 µM primers for four different regions of PD-L1 promoter were used for the qPCR analysis (Supplementary Table S1) in a QuantStudio™ 6 Flex Real-Time PCR (Thermo Fisher Scientific). Intronic regions were not considered for qPCR analysis. Relative fold-change was calculated with 2^–∆∆Ct^ using *RPL30* as a reference gene.

### Immunofluorescence

J82 and 4K5 cells treated with the respective IC_50_ doses of CM-1758 or DMSO 0.01% as a control for 48 h were fixed in 4% paraformaldehyde for 15 min at 4°C. Then, cells were washed with PBS and permeabilized with 0.1% Triton X-100 in PBS for 10 min at RT. After another wash with PBS, fixed cells were blocked with 10% horse serum for 1 h at RT, followed by the incubation with a primary antibody against 5-methylcytosine with the 10% horse serum overnight at 4°C (Supplementary Table S2). Next, after a wash with PBS, cells were incubated with the respective secondary antibody conjugated with fluorochromes in 10% horse serum at RT for 1 h (Supplementary Table S2). Finally, a mounting medium (Mowiol, Thermo Fisher Scientific) with DAPI at 1 µL/mL, for staining nuclei, was used for mounting the slides. Images were acquired with an Olympus IX51 microscope.

### Protein and histone extraction

Total protein and histones were extracted from cells treated with the IC_50_ dose of CM-1758, or DMSO-treated cells. Cells were scrapped, collected and total protein was extracted from cell pellets by adding 1:1 of lysis buffer (Hepes 40 mM, Triton X-100 2%, NaCl 200 mM, MgCl2 40 mM, EGTA 20 mM, β- glycerophosphate 80 mM and distilled water) and a mix of proteinase and phosphatase inhibitors, and phenylmethylsulfonyl fluoride (PMSF) at a final dilution of 200x, followed by a centrifugation at 16 100 g for 10 min at 4°C. The supernatant was collected and stored at -80°C until further use.

For histone extraction, 500 µL of hypotonic buffer (Tris-HCL pH=8.0 10 mM, KCl 1 mM, MgCl_2_ 1.5 mM, dithiothreitol 1 mM and distilled water), proteinase and phosphatase inhibitors, and PMSF, were added to each cell pellet and incubated for 30 min at 4°C with rotation. Then, after a 10 min centrifugation at 4°C at 10 400 g, the supernatant was discarded and 400 µL of H_2_SO_4_ at 0.2 M was added to the nucleus pellet and incubated overnight at 4°C with rotation. The samples were centrifugated at 4°C for 10 min at 10 400 g and the supernatant was transferred to another tube. Next, 132 µL of trichloroacetic acid was added and incubated for 30 min on ice. After another centrifugation, the supernatant was disregarded, and the histone pellet was washed with cold acetone. Lastly, after drying, the histone pellet was eluted in water and stored at -80°C until further use. Histones and total protein samples were quantified by Qubit™ Protein Assay Kit (ThermoFisher Scientific), according to the manufacturer’s recommendations, using a Qubit 4 Fluorometer (ThermoFisher Scientific).

### Western blot

Twenty µg or 3 µg of total protein or histones, respectively, were separated in Mini-protean TGX Stain-Free gels 4-15% (Bio-Rad, Hercules, California, USA) by sodium dodecyl sulfate-polyacrylamide gel electrophoresis (SDS-PAGE). Then, were transferred by using a Trans-Blot Turbo transfer system (Bio-Rad) into an immunoblot nitrocellulose membrane in a transfer buffer containing ethanol, distilled water, and Trans-Blot® TurboTM 5x Tranfer Buffer (Bio-Rad). After that, membranes were blocked with 5% milk in tris-buffer saline (TBS)/0.1% Tween (TBS-T) for 1 h at RT. Then, membranes were incubated with primary antibodies overnight at 4°C (Supplementary Table S2). Next, membranes were washed with TBS-T and incubated with a secondary antibody coupled with horseradish peroxidase (Supplementary Table S2) for 1 h at RT. Blots were visualized by chemiluminescence (Clarity WB ECL substrate, Bio-Rad) using a ChemiDoc XRS+ (Bio-Rad). Western blots were quantified using band densitometry analysis with ImageJ software (version 1.6.1., National Institute of Health). All antibodies and dilutions used are described in Supplementary Table S2.

### Animal care and maintenance

All the animal experiments were conducted according to the European and Spanish regulations in the field: European convention ETS 123, regarding the use and protection of vertebrate mammals used in experimentation and other scientific purposes, and Directive 2010/63/UE, Spanish Law 6/2013 and Real Decreto (R.D.) 53/2013 regarding the protection and use of animals in scientific research. Procedures involving genetically modified organisms were conducted according to the proper European and Spanish Regulations: Directive 2009/41/CE, Spanish Law 9/2003, and R.D. 178/2004. The protocols were approved by “Consejería de Medio Ambiente y Ordenación del Territorio y Sostenibilidad” (protocol number PROEX 150.8/21) and all procedures were approved by CIEMAT Animal Experimentation Ethics Committee according to external and internal biosafety and bio-ethics guidelines.

Mice were housed at the CIEMAT laboratory Animal Facility (registration number ES280790000183) and routinely screened for pathogens, following the Spanish Society for the Laboratory Animal Science (SECAL) and the Federation of European Laboratory Animal Science Associations (FELASA, Tamworth, UK) recommendations. Mice were provided with food (TEKLAD Global Diet 2918) and acidified and filtered (0.2 µm) water *ad libitum*. Mice were maintained in cages type IIL, in a maximum of 5 mice per cage. Room lighting was controlled with light/dark cycles for 12/12 h, with temperature and humidity being regulated at 21 ± 2°C and 55 ± 10%, respectively.

### Syngeneic graft mouse model

Immunocompetent double knock-out mice (DKO; *Pten*^*F/F*^, *Trp53*^*F/F*^) were developed by our group through a conventional crossing of mice from different collaborators (Netherlands Cancer Institute, Mass. Institute of Technology, Stanford University), resulting in a mixed FVB/129 S background. A syngeneic graft mouse model was generated by injecting subcutaneously cells (4K5 cell line) previously established from an aggressive mouse bladder tumor (*Pten* ^*-/-*^; *Trp53* ^*-/-*^), generated with adenoviruses expressing Cre recombinase under the control of the regulatory elements of keratin 5 (Adeno-Krt5-Cre) [52]. Three million 4K5 cells were resuspended in 100 µL of DMEM and injected subcutaneously in the right flank of 3–4-month-old male and female DKO mice. Tumor growth was monitored 3 times per week using a digital caliper and tumor volume was calculated using the formula 4π/3 x (length/2) x (width/2)^2^ [2]. After tumors reached volumes between 80-90 mm^3^, mice were randomly assigned to four groups: control (*n* = 8), anti-PD-L1 (*n* = 9), CM-1758 (*n* = 9) and CM-1758+anti-PD-L1 (*n* = 9). CM-1758 (10 mg/kg; 5 times a week) and anti-PD-L1 (avelumab; 200 µg/mouse; once a week) were injected intraperitoneally. In the combination group, a phased dosing schedule was administered. Tumor sizes were normalized by subtracting the volume calculated on the treatment initiation day. After two weeks of treatment, mice were sacrificed and tumors and whole blood were collected at the time of euthanasia. For RNA extraction, tumors were embedded in RNAlater^TM^ Stabilization solution (Thermo Fisher Scientific) at 4 °C for 24 h, and then stored at -80°C until further use. For flow cytometry, tumor tissue was embedded in DMEM and kept at 4 °C overnight to be processed and analyzed in the following day. Regarding histological analysis, tumors were fixed in 3.7-4% buffered formalin overnight at the time of the harvesting. Then, samples were embedded in paraffin and sections of 3-5 µm were stained for hematoxylin and eosin. Blood was collected in Microvette® CB 300 blood collection system with lithium heparin (Kent Scientific, Torrington, Connecticut, USA). Tubes were centrifuged at 1000 g for 10 min at 4 °C, plasma was collected and stored at -80°C until further use.

### Cell population analysis by flow cytometry

For cell population analysis, tumors were minced in small pieces and incubated with an enzymatic cocktail containing collagenase P (200 µg/mL; Roche), dispase II (800 µg/mL; Roche) and DNase I (100 µg/mL; Roche) in DMEM at 37°C, with vortex and mixing every 20 min 3-4 times. Disaggregated tissue was filtered using a 40 µm cell strainer and centrifuged at 16 100 g for 7 min. Cells were resuspended in FACS buffer (0.05% sodium azide, 0.5% bovine serum albumin and PBS) and stained with the viability dye Zombie Aqua (Biolegend), for 20 min in the dark at RT. After washing, cells were incubated with mouse FcR blocking reagent (Milteny Biotec) for 10 min in the dark at RT. Then, the antibodies described in Supplementary Table S3 were added and incubated for 30 min at 4°C. After washing, cells were fixed with 150 µL of BD Cytofix/Cytoperm™ (BD Biosciences) and acquired in the next day in a BD LSR Fortessa flow cytometer. FlowJo^TM^ 10.8.1 software (BD Biosciences) and OMIQ (Dotmatics, Boston, Massachusetts, USA) were used for data analysis.

### Cytokine analysis

Cytokine levels were analyzed using the LEGENDplex^TM^ mouse anti-virus response panel (Biolegend) according to the manufacturer’s recommendations and using a filter 96-well plate. Briefly, 25 µL of plasma was added to each well along with assay buffer and beads. The mixture was incubated for 2 h with shaking, and next wells were washed and incubated with the detection antibodies for 1 h with shaking at RT. Subsequently, SA-PE was added and incubated for 30 min at RT with shaking. Finally, samples were analyzed in CytoFLEX and CytExpert (Beckman Coulter, USA). Data was presented as fold-change in anti-PD-L1, CM-1758 and CM-1758+anti-PD-L1 groups relative to the mean value of each cytokine for control mice.

### Immunohistochemistry

Sections of formalin-fixed paraffin-embedded (FFPE) tumors were deparaffinized and incubated with hydrogen peroxide (0.3%) in methanol for 10 min. Then, antigen retrieval was performed using sodium citrate buffer (citric acid monohydrate 1.8 mM, trisodium citrate dihydrate 8.2 mM, pH=6.0) and a pressure cooker (Dako Agilent Tecnologies, Santa Clara, California, USA). Subsequently, tumor sections were blocked with 10% horse serum for 1 h at RT and incubated with the primary antibody overnight at 4°C (Supplementary Table S2). In the next day, slides were incubated with the appropriate secondary antibody for 1 h at RT. Next, the slides were incubated with the biotin-avidin-peroxidase system Vectastain Elite ABC HRP kit (Vector Labs, Newark, California, USA) for 30 min at RT, and staining was revealed with a DAB substrate kit (Vector Labs). Slides were counterstained with hematoxylin and mounted with DPX mounting media. All slides were scanned in a Pannoramic MIDI II (3DHISTECH, Budapest, Hungary) at ×20 magnification.

### Bioinformatic analyzes

For flow cytometry analysis, single and live cells were selected using the gating strategy demonstrated in Supplementary Figs. S1–4. Optimization of t-Distributed Stochastic Neighbor Embedding (opt-tSNE) was performed using OMIQ (Dotmatics) for lymphocyte and macrophage populations. Subsampling with 3000 events for each sample and 3000 interactions were used for opt-tSNE.

Regarding RNA-sequencing data, differential analyzes were performed using the DESeq2 software package (version 1.38.2) in R software [53, 54]. Normalized gene expression data were transformed into log2 scale, and the value range was adjusted for each gene. Hierarchical clustering was performed using Multi-Experiment Viewer (Mev 4.9.0). Supervised hierarchical clustering was applied using Pearson correlation and average linkage. Heatmaps were represented using SRplot (http://www.bioinformatics.com.cn/srplot). Gene set enrichment analysis (GSEA) was performed using GSEA version 4.3.2 and Molecular Signature Database (MSigDB) [55]. The h.all.v7.5.symbols.gmt (Hallmarks) gene set database was used as the gene set collection analysis. GSEA was performed using 1000 permutations and the maximum and minimum sizes for gene sets were 500 and 15, respectively. Data was represented by enrichment bubble plots, using SRplot. Venn diagrams were plotted using jvenn (https://xcmsonline.scripps.edu/lib/jvenn/example.html). Genes with a log2(foldchange) >2 for upregulated genes and <-2 downregulated genes and a *p* value adjust <0.01 were selected for venn diagram and Enrichr analysis (https://maayanlab.cloud/Enrichr/). Gene set variation analysis (GSVA) was performed using GSVA package in R software (v1.42.0) and a hallmark gene set. Data was represented by a heatmap using SRplot. We reconstructed transcriptional regulatory networks (regulons) using the R package RTN78. We investigated 1612 transcription factors (TFs) compiled from [56]. Potential associations between a regulator and all possible target genes were inferred from the expression matrix by Mutual Information and Spearman’s correlation, and permutation analysis was used to remove associations with a BH-adjusted *p* value > 0.05. Unstable associations were eliminated by bootstrap analysis (1000 resamplings, consensus bootstrap > 95%) and the weakest association in triangles of two regulators and common target genes were removed by data processing inequality (DPI) filtering (tolerance = 0.01). Regulon activity scores for all samples were calculated by two-tailed gene set enrichment analysis [56–58]. Principal component analysis (PCA) was performed using the “prcomp” command in R and the normalized matrix obtained from DeSEq2. The PCA was plotted using the PCA 3D Visualiser tool (https://prismtc.co.uk/resources/free-tools/pca-3d-visualiser).

QuPath software was used for the analysis of the necrosis areas and BrdU immunohistochemistry [59]. Briefly, the protocol for nucleus detection was optimized according to staining-intrinsic characteristics. Both BrdU and CD8 showed a clear staining and the positive cell detection tool was used to detect the positive cells using a threshold manually refined for each tumor. BrDU-positive cells are displayed as a percentage of positive cells, whereas CD8 + T cells are presented as cells per mm^2^ (cells/mm^2^). The necrosis areas were defined manually by a trained pathologist and the area value was determined using the annotation tool. The percentage of necrosis was calculated for each tumor by dividing the value of the necrosis area by the total value of tumor area and multiplying by 100.

### Statistical analysis

The in vitro studies were conducted in a minimum of 3 independent experiments. IC_50_ analyzes were performed using GraphPad Prim 8.0 software (GraphPad Software, USA). Graphics are represented by mean ± SEM. Normal distribution was evaluated using the Shapiro-Wilk test (*p* > 0.05). Data showing normal distribution were analyzed using parametric two-tailed t-test with Levene’s test for equality of variances (*p* < 0.05, variances considered not equal; *p* > 0.05, variances considered equal). Nonparametric Mann-Whitney U test was used to compare data not having a normal distribution. Immunohistochemistry results were analyzed by Fisher’s exact test. Two-tailed *p* values calculation were performed using a computer-assisted program (SPSS Version 28.0, USA). Graphics were assembled with GraphPad 8 Prism (GraphPad Software, USA). *P* values were considered statistically significant when inferior to 0.05. Significance is depicted as follows: *p < 0.05, **p < 0.01, ***p < 0.001, ****p < 0.0001 and ns > 0.05 (nonsignificant).

### Supplementary information

Supplementary Material 1

## Data Availability

The datasets discussed in this publication have been deposited in NCBI’s Gene Expression Omnibus and are accessible through GEO Series accession number GSE245122.

## References

[1] Sung H, Ferlay J, Siegel RL, Laversanne M, Soerjomataram I, Jemal A (2021). Global Cancer Statistics 2020: GLOBOCAN Estimates of Incidence and Mortality Worldwide for 36 Cancers in 185 Countries. CA Cancer J Clin.

[2] Cumberbatch MGK, Jubber I, Black PC, Esperto F, Figueroa JD, Kamat AM (2018). Epidemiology of Bladder Cancer: A Systematic Review and Contemporary Update of Risk Factors in 2018. Eur Urol.

[3] Amin MB, Edge SB, Greene FL, Byrd DR, Brookland RK, Washington MK, et al. AJCC Cancer Staging Manual. Springer International Publishing, (2018).

[4] Powles T, Bellmunt J, Comperat E, De Santis M, Huddart R, Loriot Y (2022). Bladder cancer: ESMO Clinical Practice Guideline for diagnosis, treatment and follow-up. Ann Oncol.

[5] Rhea LP, Mendez-Marti S, Kim D, Aragon-Ching JB (2021). Role of immunotherapy in bladder cancer. Cancer Treat Res Commun.

[6] Hussain SA, Birtle A, Crabb S, Huddart R, Small D, Summerhayes M (2018). From Clinical Trials to Real-life Clinical Practice: The Role of Immunotherapy with PD-1/PD-L1 Inhibitors in Advanced Urothelial Carcinoma. Eur Urol Oncol.

[7] Hanahan D (2022). Hallmarks of Cancer: New Dimensions. Cancer Discov.

[8] Baylin SB, Jones PA (2016). Epigenetic Determinants of Cancer. Cold Spring Harb Perspect Biol.

[9] Audia JE, Campbell RM (2016). Histone Modifications and Cancer. Cold Spring Harb Perspect Biol.

[10] Miranda Furtado CL, Dos Santos Luciano MC, Silva Santos RD, Furtado GP, Moraes MO, Pessoa C (2019). Epidrugs: targeting epigenetic marks in cancer treatment. Epigenetics.

[11] Ramaiah MJ, Tangutur AD, Manyam RR (2021). Epigenetic modulation and understanding of HDAC inhibitors in cancer therapy. Life Sci.

[12] Rubio C, Avendaño-Ortiz J, Ruiz-Palomares R, Karaivanova V, Alberquilla O, Sánchez-Domínguez R (2022). Toward Tumor Fight and Tumor Microenvironment Remodeling: PBA Induces Cell Cycle Arrest and Reduces Tumor Hybrid Cells’ Pluripotency in Bladder Cancer. Cancers (Basel).

[13] Wang SC, Wang ST, Liu HT, Wang XY, Wu SC, Chen LC (2017). Trichostatin A induces bladder cancer cell death via intrinsic apoptosis at the early phase and Sp1‑survivin downregulation at the late phase of treatment. Oncol Rep..

[14] Burke B, Eden C, Perez C, Belshoff A, Hart S, Plaza-Rojas L (2020). Inhibition of Histone Deacetylase (HDAC) Enhances Checkpoint Blockade Efficacy by Rendering Bladder Cancer Cells Visible for T Cell-Mediated Destruction. Front Oncol.

[15] Li X, Su X, Liu R, Pan Y, Fang J, Cao L (2021). HDAC inhibition potentiates anti-tumor activity of macrophages and enhances anti-PD-L1-mediated tumor suppression. Oncogene.

[16] Truong AS, Zhou M, Krishnan B, Utsumi T, Manocha U, Stewart KG (2021). Entinostat induces antitumor immune responses through immune editing of tumor neoantigens. J Clin Invest.

[17] Lodewijk I, Nunes SP, Henrique R, Jerónimo C, Dueñas M, Paramio JM (2021). Tackling tumor microenvironment through epigenetic tools to improve cancer immunotherapy. Clin Epigene.

[18] Rabal O, San José-Enériz E, Agirre X, Sánchez-Arias JA, de Miguel I, Ordoñez R (2021). Design and Synthesis of Novel Epigenetic Inhibitors Targeting Histone Deacetylases, DNA Methyltransferase 1, and Lysine Methyltransferase G9a with In Vivo Efficacy in Multiple Myeloma. J Med Chem.

[19] Huang KC, Chiang SF, Chen WT, Chen TW, Hu CH, Yang PC (2020). Decitabine Augments Chemotherapy-Induced PD-L1 Upregulation for PD-L1 Blockade in Colorectal Cancer. Cancers (Basel).

[20] Woods DM, Sodré AL, Villagra A, Sarnaik A, Sotomayor EM, Weber J (2015). HDAC Inhibition Upregulates PD-1 Ligands in Melanoma and Augments Immunotherapy with PD-1 Blockade. Cancer Immunol Res.

[21] Zang J, Ye K, Fei Y, Zhang R, Chen H, Zhuang G (2021). Immunotherapy in the Treatment of Urothelial Bladder Cancer: Insights From Single-Cell Analysis. Front Oncol.

[22] Singh M, Kumar V, Sehrawat N, Yadav M, Chaudhary M, Upadhyay SK (2022). Current paradigms in epigenetic anticancer therapeutics and future challenges. Semin Cancer Biol.

[23] Hoffmann MJ, Meneceur S, Hommel K, Schulz WA, Niegisch G (2021). Downregulation of Cell Cycle and Checkpoint Genes by Class I HDAC Inhibitors Limits Synergism with G2/M Checkpoint Inhibitor MK-1775 in Bladder Cancer Cells. Genes (Basel).

[24] Wang C, Hamacher A, Petzsch P, Köhrer K, Niegisch G, Hoffmann MJ (2020). Combination of Decitabine and Entinostat Synergistically Inhibits Urothelial Bladder Cancer Cells via Activation of FoxO1. Cancers (Basel).

[25] Li QQ, Hao JJ, Zhang Z, Hsu I, Liu Y, Tao Z (2016). Histone deacetylase inhibitor-induced cell death in bladder cancer is associated with chromatin modification and modifying protein expression: A proteomic approach. Int J Oncol.

[26] Kaletsch A, Pinkerneil M, Hoffmann MJ, Jaguva Vasudevan AA, Wang C, Hansen FK (2018). Effects of novel HDAC inhibitors on urothelial carcinoma cells. Clin Epigenetics.

[27] Moore LD, Le T, Fan G (2013). DNA methylation and its basic function. Neuropsychopharmacology.

[28] Witjes JA, Bruins HM, Cathomas R, Compérat EM, Cowan NC, Gakis G (2021). European Association of Urology Guidelines on Muscle-invasive and Metastatic Bladder Cancer: Summary of the 2020 Guidelines. Eur Urol.

[29] Segovia C, San José-Enériz E, Munera-Maravilla E, Martínez-Fernández M, Garate L, Miranda E (2019). Inhibition of a G9a/DNMT network triggers immune-mediated bladder cancer regression. Nat Med.

[30] Shen Y, Liu L, Wang M, Xu B, Lyu R, Shi YG (2021). TET2 Inhibits PD-L1 Gene Expression in Breast Cancer Cells through Histone Deacetylation. Cancers (Basel).

[31] Bannister AJ, Kouzarides T (2011). Regulation of chromatin by histone modifications. Cell Res.

[32] Chen X, Xu R, He D, Zhang Y, Chen H, Zhu Y (2021). CD8(+) T effector and immune checkpoint signatures predict prognosis and responsiveness to immunotherapy in bladder cancer. Oncogene.

[33] Sprooten J, De Wijngaert P, Vanmeerbeerk I, Martin S, Vangheluwe P, Schlenner S (2020). Necroptosis in Immuno-Oncology and Cancer Immunotherapy. Cells.

[34] Seitz C, Gupta A, Shariat SF, Matsumoto K, Kassouf W, Walton TJ (2010). Association of tumor necrosis with pathological features and clinical outcome in 754 patients undergoing radical nephroureterectomy for upper tract urothelial carcinoma: an international validation study. J Urol.

[35] Chow A, Perica K, Klebanoff CA, Wolchok JD (2022). Clinical implications of T cell exhaustion for cancer immunotherapy. Nat Rev Clin Oncol.

[36] Niu Y, Chen J, Qiao Y (2022). Epigenetic Modifications in Tumor-Associated Macrophages: A New Perspective for an Old Foe. Front Immunol.

[37] Boyerinas B, Jochems C, Fantini M, Heery CR, Gulley JL, Tsang KY (2015). Antibody-Dependent Cellular Cytotoxicity Activity of a Novel Anti-PD-L1 Antibody Avelumab (MSB0010718C) on Human Tumor Cells. Cancer Immunol Res.

[38] Gubin MM, Esaulova E, Ward JP, Malkova ON, Runci D, Wong P (2018). High-Dimensional Analysis Delineates Myeloid and Lymphoid Compartment Remodeling during Successful Immune-Checkpoint Cancer Therapy. Cell.

[39] Martinez VG, Munera-Maravilla E, Bernardini A, Rubio C, Suarez-Cabrera C, Segovia C (2019). Epigenetics of Bladder Cancer: Where Biomarkers and Therapeutic Targets Meet. Front Genet.

[40] He M, He Q, Cai X, Chen Z, Lao S, Deng H (2021). Role of lymphatic endothelial cells in the tumor microenvironment-a narrative review of recent advances. Transl Lung Cancer Res.

[41] Yang D, Liu J, Qian H, Zhuang Q (2023). Cancer-associated fibroblasts: from basic science to anticancer therapy. Exp Mol Med.

[42] Martins-Lima C, Chianese U, Benedetti R, Altucci L, Jerónimo C, Correia MP (2022). Tumor microenvironment and epithelial-mesenchymal transition in bladder cancer: Cytokines in the game?. Front Mol Biosci.

[43] Ivashkiv LB (2018). IFNγ: signalling, epigenetics and roles in immunity, metabolism, disease and cancer immunotherapy. Nat Rev Immunol.

[44] Quaranta V, Schmid MC (2019). Macrophage-Mediated Subversion of Anti-Tumour Immunity. Cells.

[45] Aldinucci D, Borghese C, Casagrande N (2020). The CCL5/CCR5 Axis in Cancer Progression. Cancers (Basel).

[46] Huang CP, Liu LX, Shyr CR (2020). Tumor-associated Macrophages Facilitate Bladder Cancer Progression by Increasing Cell Growth, Migration, Invasion and Cytokine Expression. Anticancer Res.

[47] Li Y, Chen X, Li D, Yang Z, Bai Y, Hu S (2021). Identification of prognostic and therapeutic value of CC chemokines in Urothelial bladder cancer: evidence from comprehensive bioinformatic analysis. BMC Urol.

[48] Fridman WH, Pagès F, Sautès-Fridman C, Galon J (2012). The immune contexture in human tumours: impact on clinical outcome. Nat Rev Cancer.

[49] Tham SM, Ng KH, Pook SH, Esuvaranathan K, Mahendran R (2011). Tumor and microenvironment modification during progression of murine orthotopic bladder cancer. Clin Dev Immunol.

[50] Earl J, Rico D, Carrillo-de-Santa-Pau E, Rodríguez-Santiago B, Méndez-Pertuz M, Auer H (2015). The UBC-40 Urothelial Bladder Cancer cell line index: a genomic resource for functional studies. BMC Genom.

[51] Livak KJ, Schmittgen TD (2001). Analysis of relative gene expression data using real-time quantitative PCR and the 2(-Delta Delta C(T)) Method. Methods.

[52] Ramírez A, Bravo A, Jorcano JL, Vidal M (1994). Sequences 5’ of the bovine keratin 5 gene direct tissue- and cell-type-specific expression of a lacZ gene in the adult and during development. Differentiation.

[53] Team RC. R: A language and environment for statistical computing. R Foundation for Statistical Computing (2022).

[54] Love MI, Huber W, Anders S (2014). Moderated estimation of fold change and dispersion for RNA-seq data with DESeq2. Genome Biol.

[55] Subramanian A, Tamayo P, Mootha VK, Mukherjee S, Ebert BL, Gillette MA (2005). Gene set enrichment analysis: a knowledge-based approach for interpreting genome-wide expression profiles. Proc Natl Acad Sci USA.

[56] Lambert SA, Jolma A, Campitelli LF, Das PK, Yin Y, Albu M (2018). The Human Transcription Factors. Cell.

[57] Robertson AG, Kim J, Al-Ahmadie H, Bellmunt J, Guo G, Cherniack AD (2017). Comprehensive Molecular Characterization of Muscle-Invasive Bladder. Cancer Cell.

[58] Lindskrog SV, Prip F, Lamy P, Taber A, Groeneveld CS, Birkenkamp-Demtröder K (2021). An integrated multi-omics analysis identifies prognostic molecular subtypes of non-muscle-invasive bladder cancer. Nat Commun.

[59] Bankhead P, Loughrey MB, Fernández JA, Dombrowski Y, McArt DG, Dunne PD (2017). QuPath: Open source software for digital pathology image analysis. Sci Rep.

