# Supplementary material for: Modulation of tumor microenvironment by targeting histone acetylation in bladder cancer
Source: Cell Death Discov. 2024 Jan 4;10:1. doi: 10.1038/s41420-023-01786-3 (PMC10764810; doi:10.1038/s41420-023-01786-3)
Supplement: Supplementary file 1 — Supplementary Material 1 [file 41420_2023_1786_MOESM1_ESM.docx]

**Supplementary Information**

**Supplementary Figures**


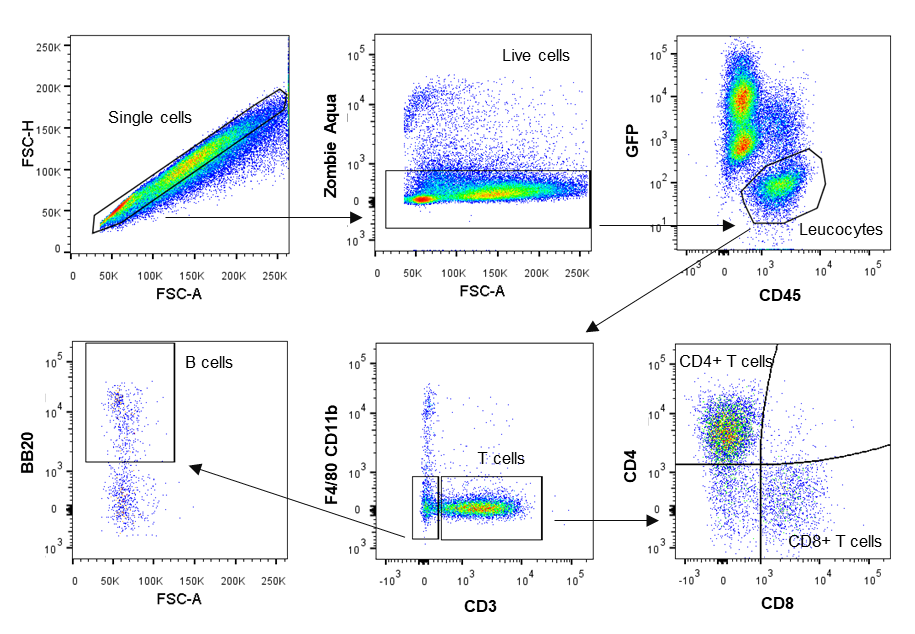


**Figure S1**. Representative gating strategy for tumor T (CD4+ and CD8+) and B cells gated as single cells, live cells, GFP negative CD45 positive, F4/80 CD11b negative CD3 positive, CD4 and CD8 positive for T cells and F4/80 CD11b negative CD3 negative.


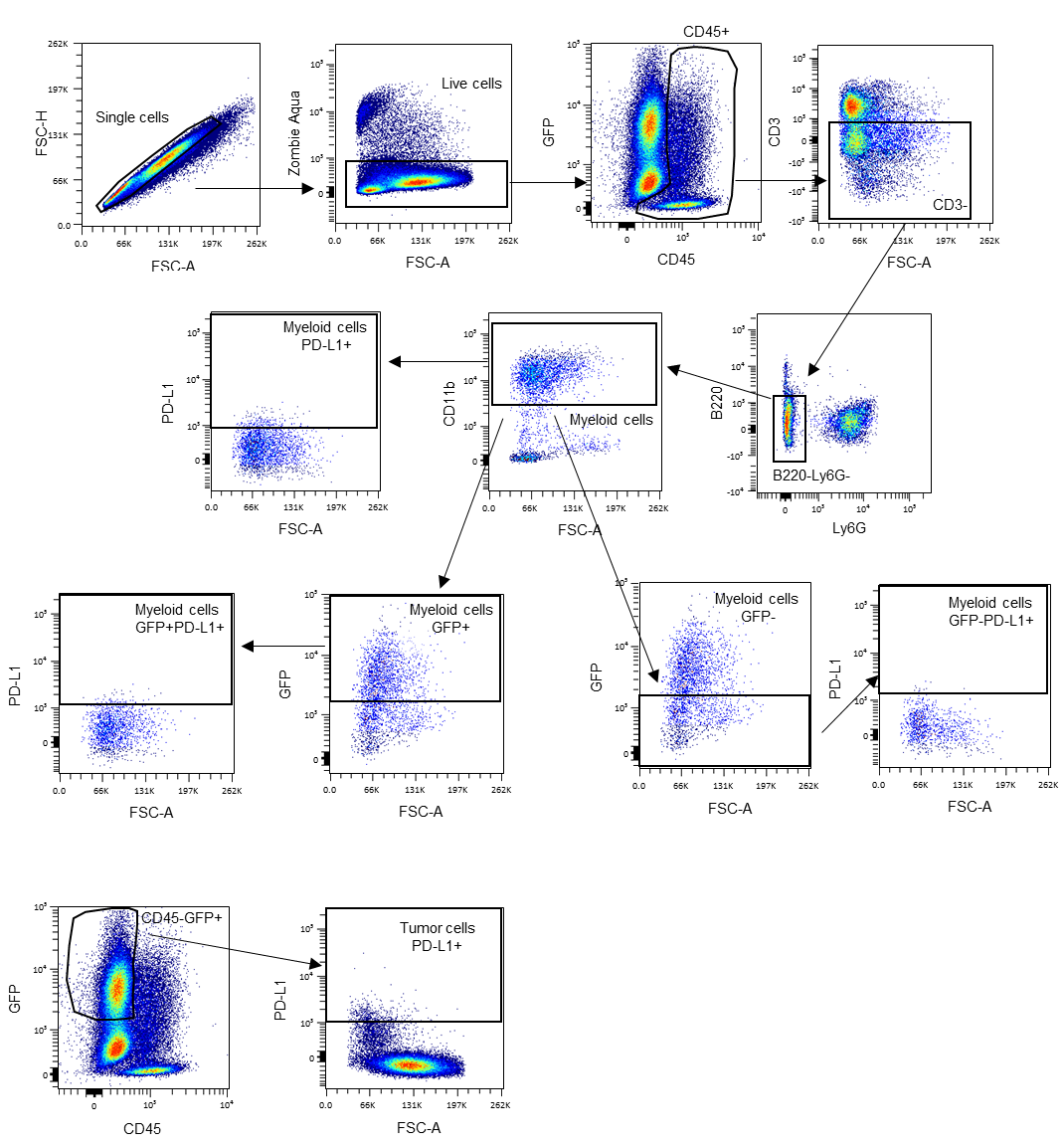


**Figure S2**. Representative gating strategy for tumor myeloid cells gated as single cells, live cells, CD45 positive, CD3, B220 and Ly6G negative, CD11b positive, GFP positive or negative. Tumor cells were gated using single cells, live cells, CD45 negative, GFP positive. PD-L1 levels were evaluated in both myeloid and tumor cells.


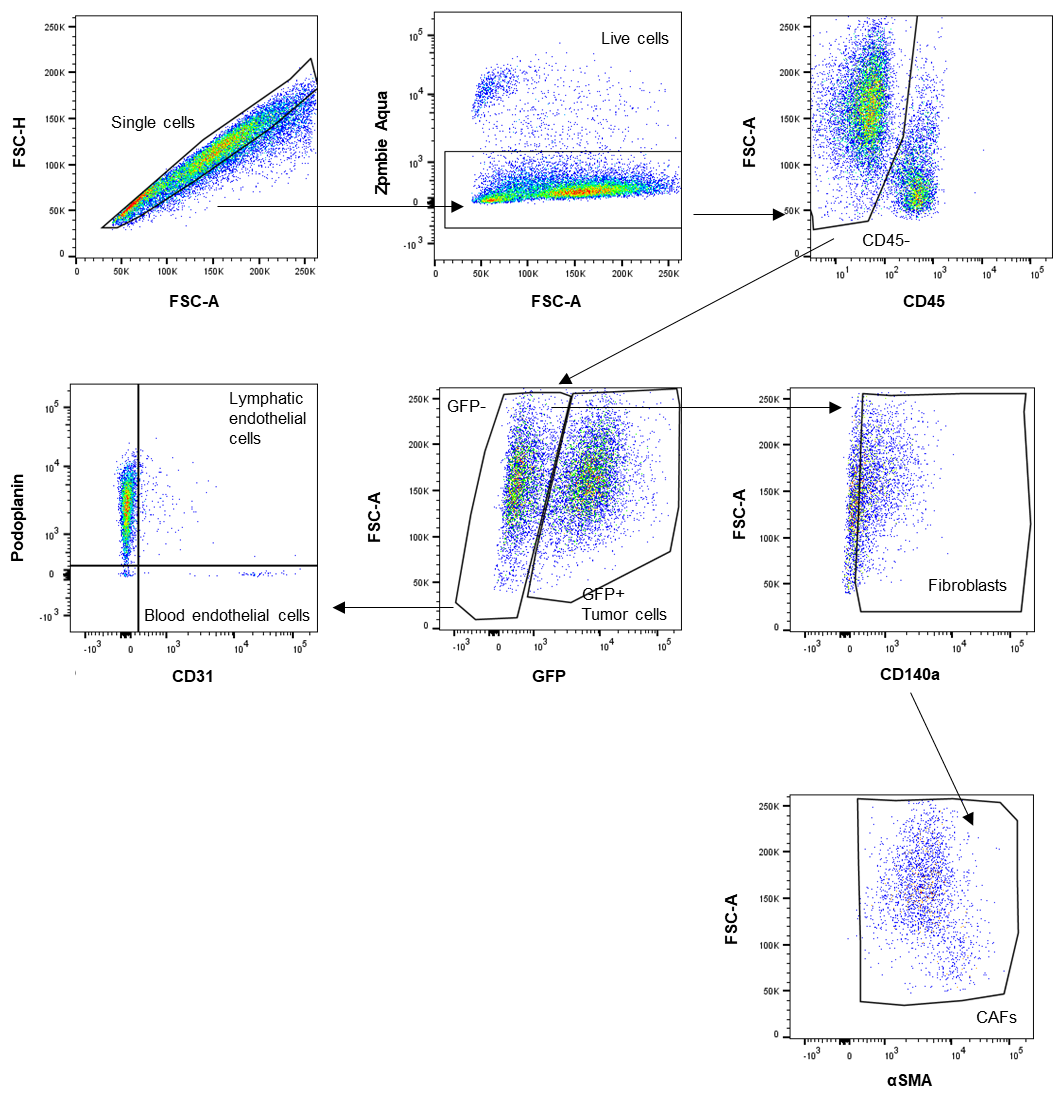


**Figure S3**. Representative gating strategy for fibroblasts, lymphatic and blood endothelial cells as single cells, live cells, CD45 negative, GFP negative, CD31 positive for blood endothelial cells, CD31 positive podoplanin positive for lymphatic endothelial cells. Fibroblasts were gated as GFP negative, CD140a positive. Cancer-associated fibroblasts (CAFs) were defined as GFP negative, CD140a positive and αSMA positive.


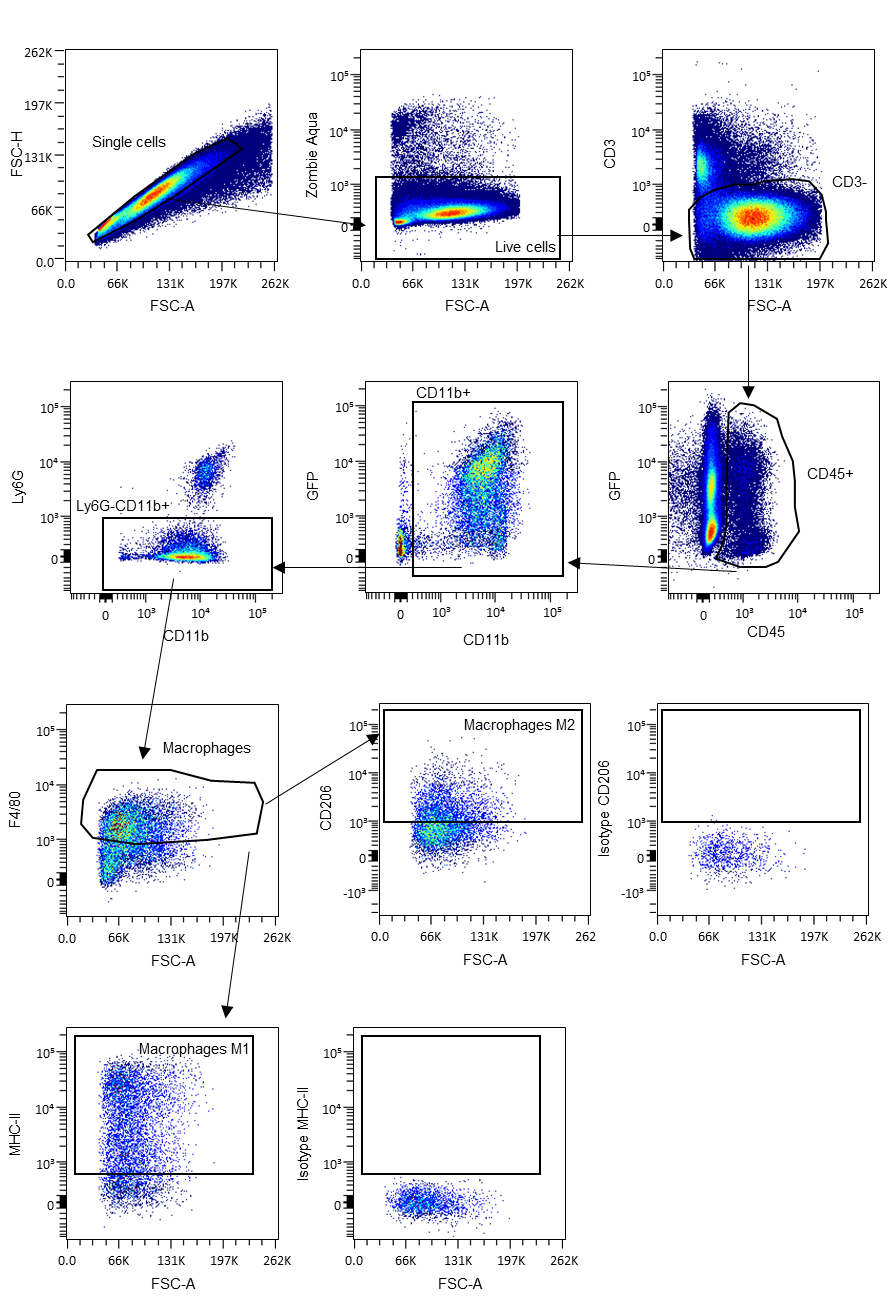


**Figure S4**. Representative gating strategy for tumor macrophages and respective status gated as single cells, live cells, CD3 negative, CD45 positive, CD11b positive, Ly6G negative, F4/80 positive, CD206 positive for M2-like macrophages, MHC-II positive for M1-like macrophages with respective isotype control.


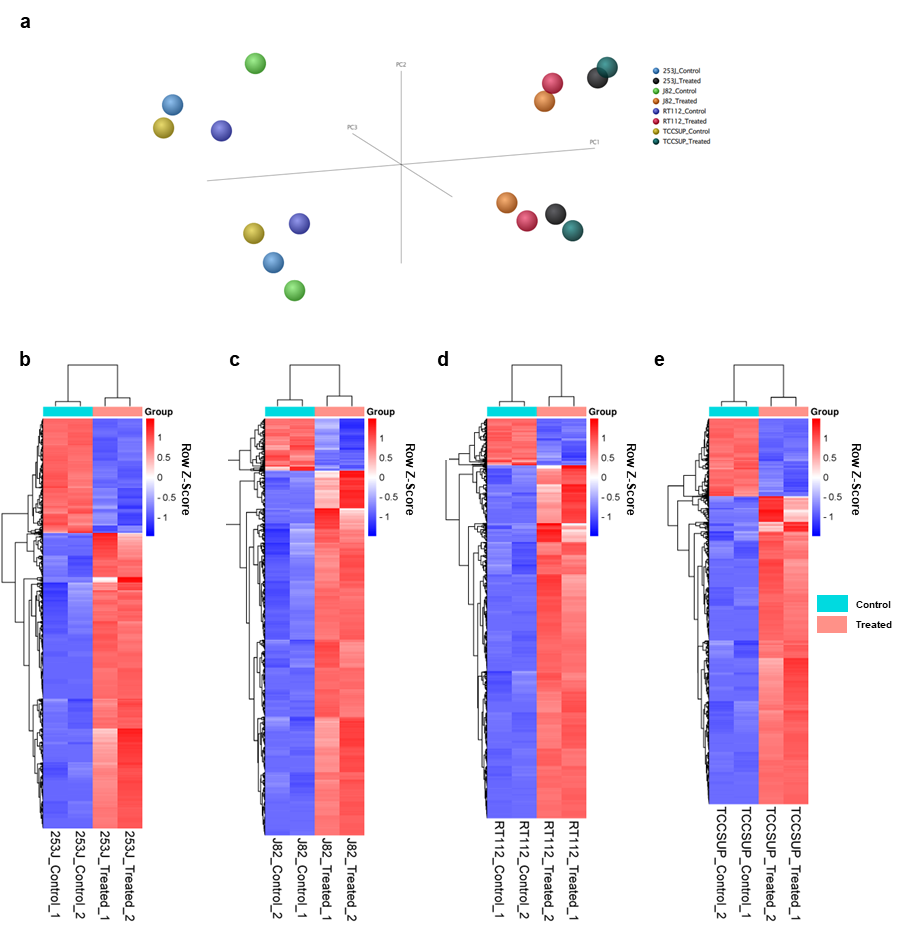


**Figure S5.** (a) Principal component analysis (PCA) graph of 253J, J82, RT112 and TCCSUP together showing the aggrupation of the conditions control vs. treated for each cell line. (b-e) Individual heatmaps of (b) 253J, (c) J82, (d) RT112 and (e) TCCSUP of the dysregulated genes between untreated control vs. treated cells. Genes and groups (control and treated) are hierarchically clustered. Control denotes untreated cells, whereas treated refers to CM-1758 treated cells. Treatment with CM-1758 was given for 48 h with IC_50_ dose calculated for each cell line. Two independent experiments were included for each cell line and condition.


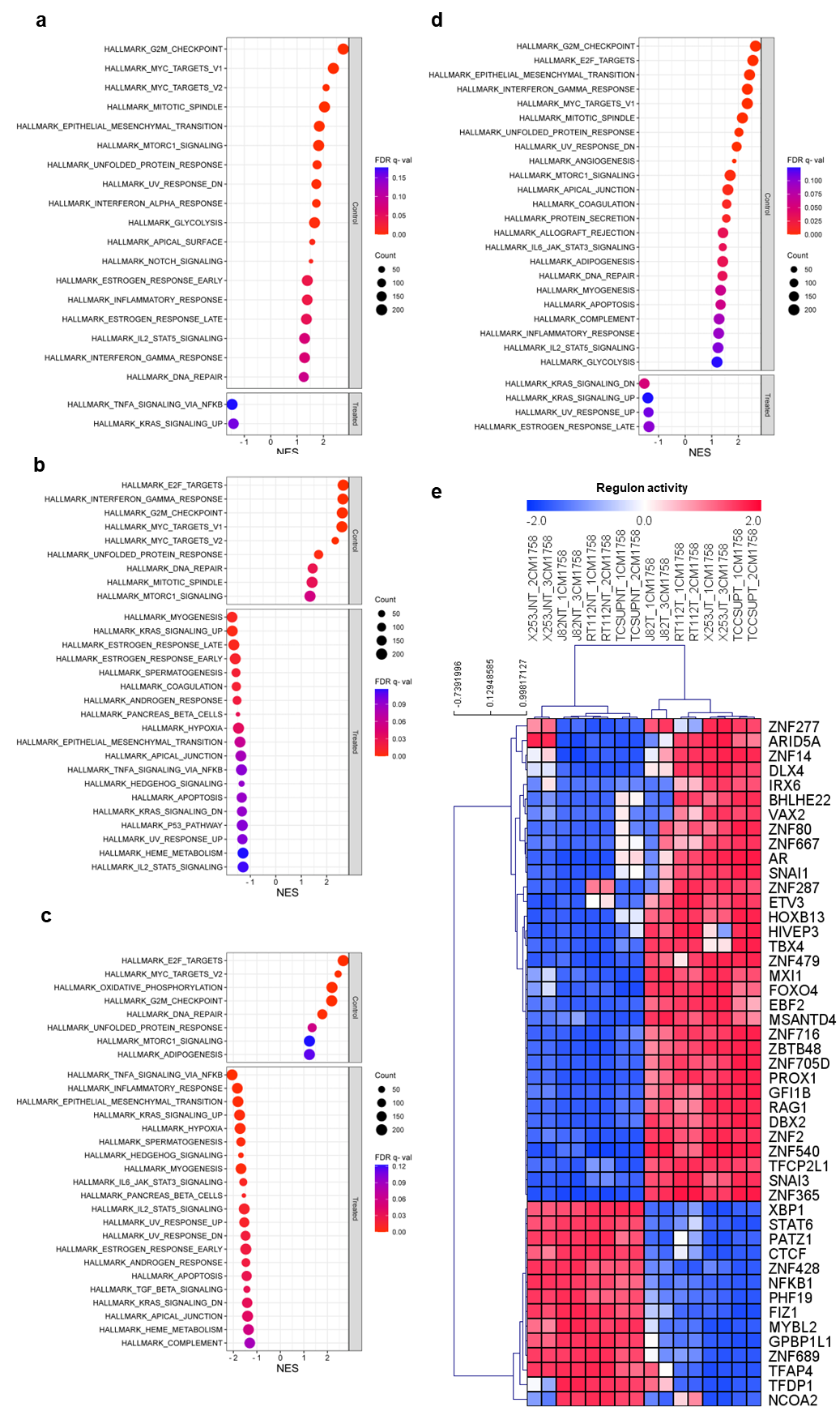


**Figure S6.** Enrichment bubble plot of (a) 253J, (b) J82, (c) RT112 and (d) TCCSUP cells for the hallmark gene signatures of GSEA analysis comparing untreated control cells vs. treated cells with IC_50_ dose of CM-1758 for 48 h. NES – Normalized enrichment score; FDR q-value – False discovery rate q-value; (e) Regulon activity profile for transcription factors hierarchically clustered. Control denotes untreated cells, whereas treated refers to CM-1758 treated cells. Cells were treated the IC_50_ dose of CM-1758 for 48 h.


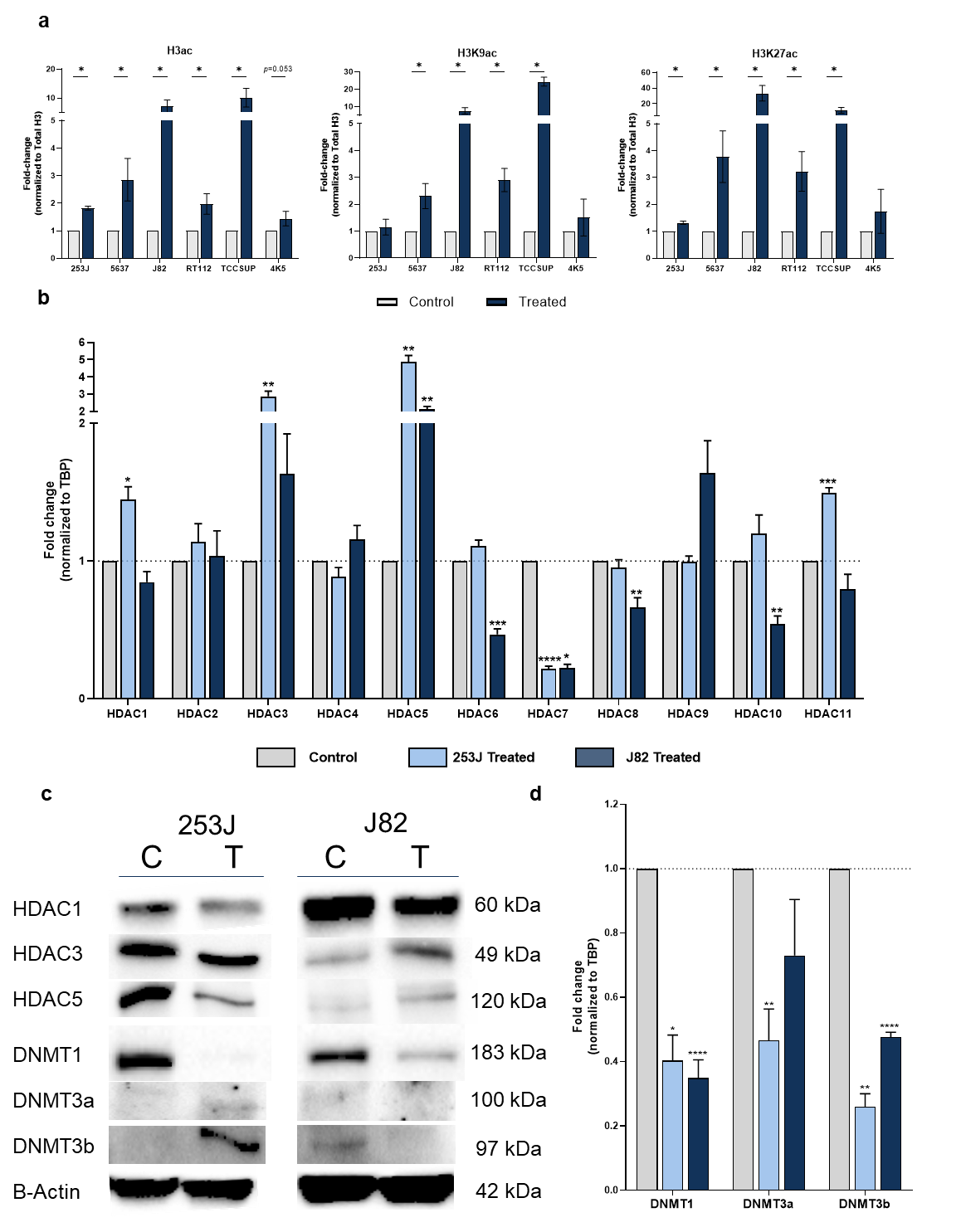


**Figure S7.** (a) Quantification of H3ac, H3K9ac and H3K27ac levels by western blot respect to total H3. Values represented as fold-change respective to control non-treated cells. (b) Relative expression by RT-qPCR of HDAC1 through 11 and (c) Western blot analysis of HDAC1, HDAC3, HDAC5, DNMT1, DNMT3a and DNMT3b with respective β-actin levels for 253J and J82 cells after treatment with CM-1758. (d) DNMT1, DNMT3a and DNMT3b respect to TBP for 253J and J82 cells after treatment with CM-1758. Cells were treated with CM-1758 for 48 h with the IC_50_ dose calculated for each cell line. Data shown are the mean of ≥3 experiments ± SEM. P-values are represented as ns – not significant, *<0.05, **<0.01 and ***<0.001.

**
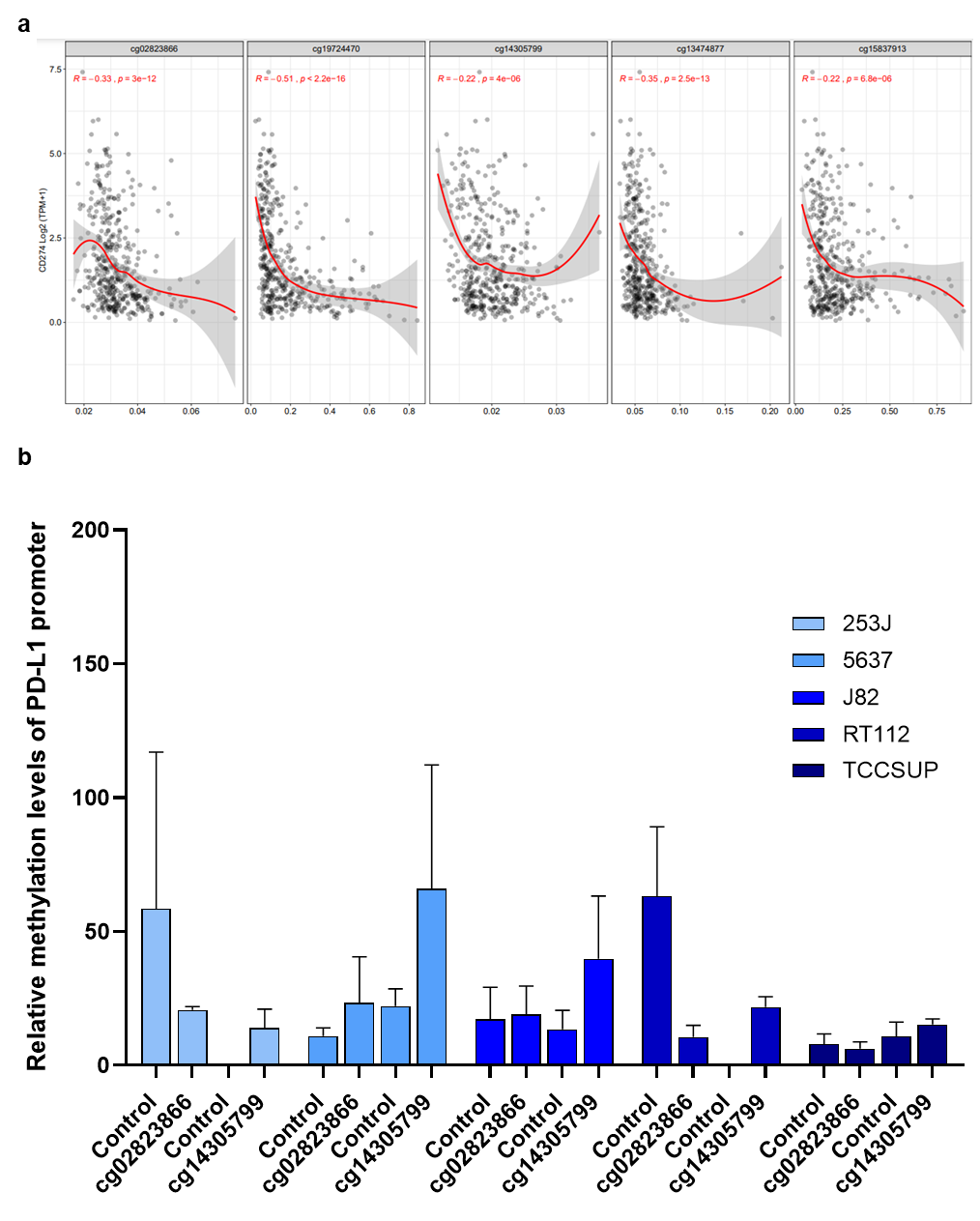
**

**Figure S8**. (a) CGs associated with PD-L1 expression using the Shiny Methylation Analysis Resource Tool (SMART). Two CGs cg02823866 and cg14305799 were selected for qMSP since they were localized inside a PD-L1 promoter CpG island and it was possible to design primers to specifically amplify these CGs regions of the promoter (b) Relative methylation levels of cg02823866 and cg14305799 in PD-L1 promoter normalized for β-actin x 1000 for easier tabulation. Data shown are the mean of ≥3 experiments ± SEM.

**
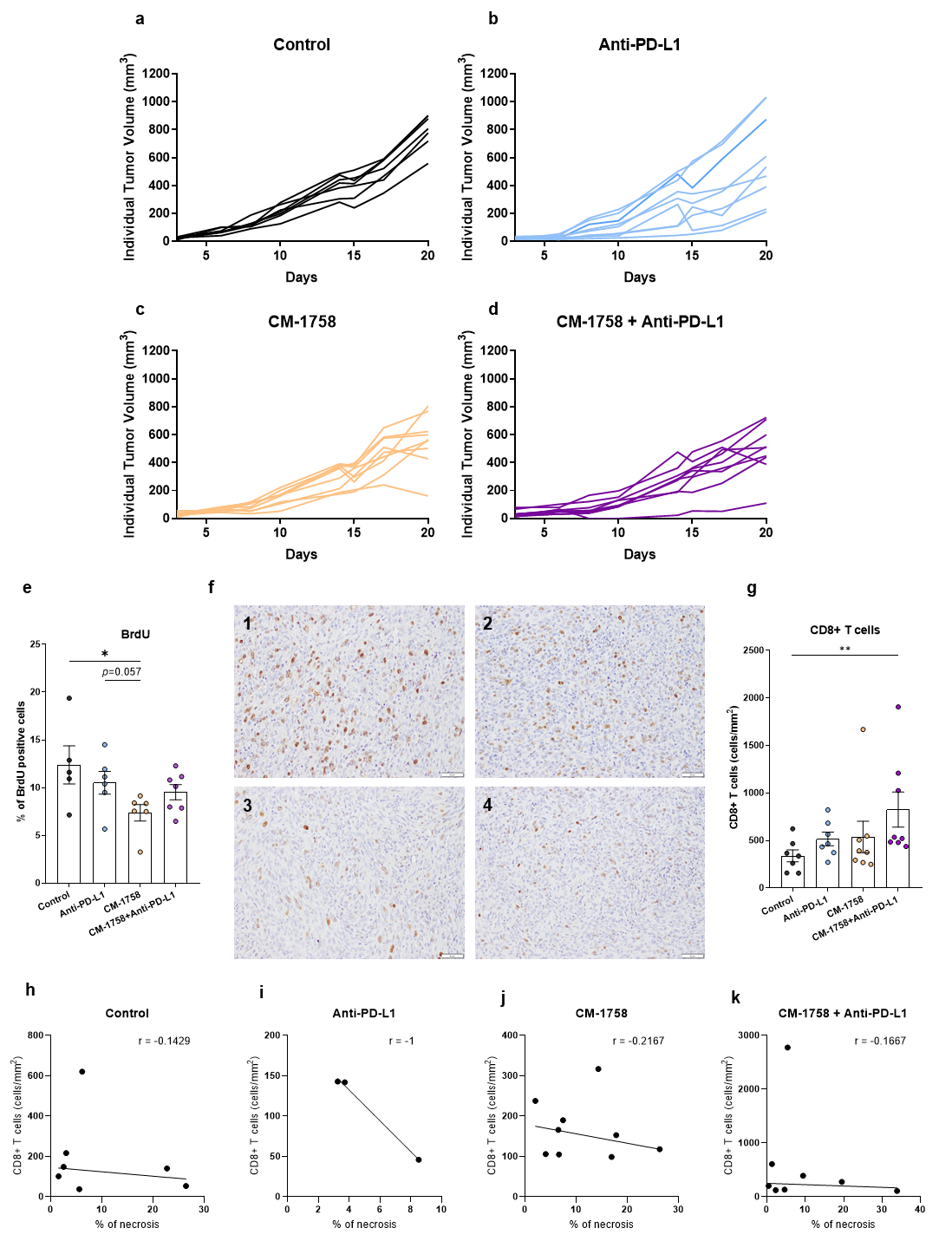
**

**Figure S9**. (a-d) Individual tumor growth curves respect to the day of 4K5 cells injection of (a) control, (b) anti-PD-L1, (c) CM-1758 and (d) CM-1758+anti-PD-L1. (e) Immunohistochemistry quantification of BrdU positive cells in the different treatment groups presented as percentage of positive cells. (f1-4) Representative immunohistochemistry staining of BrdU in each treatment group included in the study (1 – control; 2 – anti-PD-L1; 3 – CM-1758 and 4 – CM-1758 + anti-PD-L1. (g) Immunohistochemistry quantification of CD8+ T positive cells in the different treatment groups presented as cells/mm^2^. (h-k) Spearman correlation analysis of CD8+ T cells and percentage of necrosis in (h) control, (i) anti-PD-L1, (j) CM-1758 and (k) CM-1758+anti-PD-L1 with respective Spearman's rank correlation coefficient (r). Graphs show individual values as the mean ± SEM. P-values are represented as ns – not significant, *<0.05 and **<0.01.

**
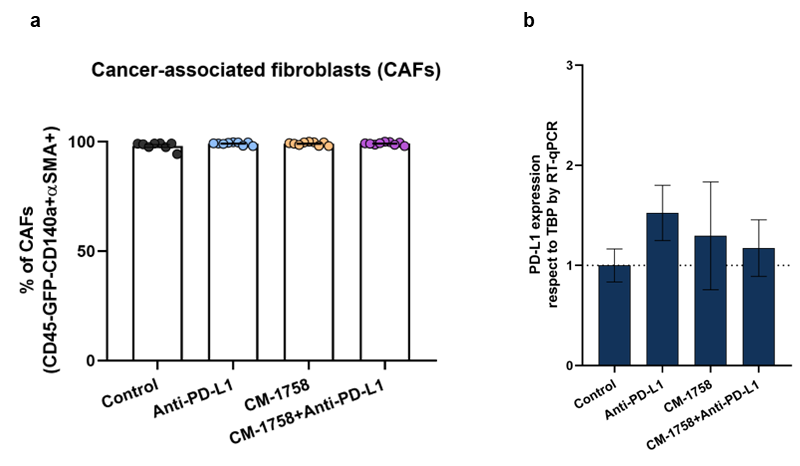
**

**Figure S10**. (a) Percentage of cancer-associated fibroblasts (CAFs) positive for α-SMA present in the tumor microenvironment gated in CD140a+ cells. (b) Relative expression by RT-qPCR of PD-L1 in respect to TBP in tumors from the groups control, anti-PD-L1, CM-1758 and CM-1758+anti-PD-L1. Graphs show individual values as the mean ± SEM. P-values are represented as ns – not significant.

**Supplementary Tables**

**Table S1.** Primers’ sequences for RT-qPCR, PD-L1 CUT&RUN and PD-L1 methylation (qMSP). The h before the primer’s name denotes human sequence and m mouse sequence. F – Forward; R – Reverse.

| Gene | Primer sequence (5’-3’) | | PCR product size |
| --- | --- | --- | --- |
| RT-qPCR | | | |
| *hPD-L1* | F | GGCATCCAAGATACAAACTCAA | 76 bp |
|  | R | CAGAAGTTCCAATGCTGGATTA |  |
| *hHDAC1* | F | CGAATCCGCATGACTCATAA | 93 bp |
|  | R | CATCTCCTCAGCATTGGCTT |  |
| *hHDAC2* | F | ATGGCGTACAGTCAAGGAGG | 102 bp |
|  | R | ATGAGGCTTCATGGGATGAC |  |
| *hHDAC3* | F | GCAAGGCTTCACCAAGAGTC | 92 bp |
|  | R | CTGTGTAACGCGAGCAGAAC |  |
| *hHDAC4* | F | CGTGGAAATTTTGAGCCATT | 98 bp |
|  | R | CTGGTCTCGGCCAGAAAGT |  |
| *hHDAC5* | F | GGGAACCATCCTTGGAAATC | 97 bp |
|  | R | GAACTGGGCATGGCTCTTG |  |
| *hHDAC6* | F | CCGGAGGGTCCTTATCGTAG | 100 bp |
|  | R | GCGGTGGATGGAGAAATAGA |  |
| *hHDAC7* | F | CTCTCTGTGGGCATCCTGG | 87 bp |
|  | R | GGTTCCAGAGCCTTAGAGATTC |  |
| *hHDAC8* | F | ATACTTGACCGGGGTCATCC | 101 bp |
|  | R | GCGTGATTTCCAGCACATAA |  |
| *hHDAC9* | F | GCAGAGGAAGAGCTTCAGGG | 79 bp |
|  | R | TGTCGCTCCTAGTGCTGTTG |  |
| *hHDAC10* | F | TGAACATCAGGGGCAAGGAG | 92 bp |
|  | R | GATGCAGCTCAGGAAACCAC |  |
| *hHDAC11* | F | GCACACGAGGCGCTATCTTA | 94 bp |
|  | R | AAGGAAGTTGGGGAGGAAGA |  |
| *hDNMT1* | F | CAAACCCCTTTCCAAACCTC | 86 bp |
|  | R | TAATCCTGGGGCTAGGTGAA |  |
| *hDNMT3a* | F | CCTGAAGCCTCAAGAGCAGT | 94 bp |
|  | R | TGGTCTCCTTCTGTTCTTTGC |  |
| *hDNMT3b* | F | AAGCGACAGGCTATGTCTGG | 90 bp |
|  | R | CAGTGCCACCAGTTTGTCTG |  |
| *hTBP* | F | AGTGAAGAACAGTCCAGACTG | 194 bp |
|  | R | CCAGGAAATAACTCTGGCTCAT |  |
| *mPD-L1* | F | GTTGTTCCTCATTGTAGTGTCCA | 77 bp |
|  | R | CACATTTCTCCACATCTAGCATTC |  |
| *mTBP* | F | GGGAGAATCATGGACCAGAA | 90 bp |
|  | R | GATGGGAATTCCAGGAGTCA |  |
| qMSP | | | |
| *hPD-L1* cg02823866 | F | AAAGTTTTCGTCGATTTTATCG | 116 bp |
|  | R | ACTCAACGTTACGCCAAACC |  |
| *hPD-L1* cg14305799 | F | CGTTTCGCGCGGTTTTAG | 150 bp |
|  | R | ACCCTCCGTCCTAAAATACCCAA |  |
| *hACTB* | F | TGGTGATGGAGGAGGTTTAGTAAG | 132 bp |
|  | R | ACCAATAAAACCTACTCCTCCCTTAA |  |
| CUT&RUN | | | |
| *hPD-L1* Region 1 | F | ACTGAAAGCTTCCGCCGATT | 74 bp |
|  | R | CCCAAGGCAGCAAATCCAGT |  |
| *hPD-L1* Region 2 | F | GGGGGACGCCTTTCTGATAA | 89 bp |
|  | R | AAGCCAACATCTGAACGCAC |  |
| *hPD-L1* Region 3 | F | CTTTGGCGGATCACTTGAGG | 67 bp |
|  | R | TATCCCGCGCTGAACTTCTA |  |
| *hPD-L1* Region 4 | F | GCTGGGCCCAAACCCTATT | 62 bp |
|  | R | TTTGGCAGGAGCATGGAGTT |  |

**Table S2**. Primary and secondary antibodies used for western blot, immunofluorescence, immunohistochemistry.

| Western Blot | | | | |
| --- | --- | --- | --- | --- |
| Primary antibody | Dilution | Secondary antibody | Company | |
| H3ac (polyclonal) | 1:1000 | Anti-rabbit | Millipore  06-599 | |
| H3K9ac (C5B11) | 1:1000 | Anti-rabbit | Cell Signaling  #9649 | |
| H3K27ac (polyclonal) | 1:1000 | Anti-rabbit | Cell Signaling  #4353 | |
| Total H3 (96C10) | 1:10 000 | Anti-mouse | Cell Signaling  #3638 | |
| HDAC1  (5C11) | 1:1000 | Anti-mouse | Sigma  SAB1400121 | |
| HDAC3  (Y415) | 1:750 | Anti-rabbit | Abcam  32369 | |
| HDAC5  (B-11) | 1:500 | Anti-mouse | Santa Cruz  sc-133106 | |
| DNMT1 (60B12201) | 1:500 | Anti-mouse | Abcam  176145 | |
| DNMT3a (D23G1) | 1:250 | Anti-rabbit | Cell Signaling  #3598 | |
| DNMT3b (polyclonal) | 1:500 | Anti-rabbit | Abcam  2851 | |
| β-actin (polyclonal) | 1:10 000 | Anti-mouse | Abcam  8227 | |
| Immunofluorescence | | | | |
| Primary antibody | Dilution | Secondary antibody | Company | |
| 5-methylcytosine  (33D3) | 1:100 | Anti-mouse | Sigma  MABE146 | |
| Immunohistochemistry | | | | |
| Primary antibody | Dilution | Secondary antibody | Company | |
| BrdU (BMC9318) | 1:10 | Anti-mouse | Roche  11170376001 | |
| CD8  (EPR21769) | 1:400 | Anti-rabbit | Abcam  ab217344 | |
| Ki67  (polyclonal) | 1:250 | Anti-rabbit | Abcam  ab15580 | |
| Secondary antibodies | | | | |
| Antibody | Conjugate | Technique | Dilution | Company |
| IgG mouse  (polyclonal) | Peroxidase | Western blot | 1:10 000 | Jackson  715-035-151 |
| IgG rabbit (polyclonal) | Peroxidase | Western blot | 1:10 000 | Amersham  NA 934 |
| IgG mouse  (polyclonal) | Alexa Fluor 594 | Immunofluorescence | 1:1000 | Invitrogen  A11005 |
| IgG mouse (polyclonal) | Biotin | Immunohistochemistry | 1:1000 | Jackson  715-065-151 |
| IgG rabbit (polyclonal) | Biotin | Immunohistochemistry | 1:1000 | Jackson  711-065-152 |

**Table S3**. Antibodies used for cell population characterization by flow cytometry.

| Epitope | Fluorochrome | Company | Reference |
| --- | --- | --- | --- |
| CD45 (30-F11) | PerCP | Biolegend | 103130 |
| CD3 (145-2C11) | PE | BD | 553064 |
| CD3 (17A2) | APC-Cy7 | Biolegend | 100222 |
| CD8a (53-6.7) | APC-Cy7 | Biolegend | 100714 |
| CD4 (RM4-5) | BV711 | Biolegend | 100550 |
| CD45R/B220 (RA3-6B2) | PEFire700 | Biolegend | 103280 |
| F4/80 (BM8) | APC | Biolegend | 123116 |
| F4/80 (BM8) | BV605 | Biolegend | 123149 |
| CD11b (M1/70) | A647 | Biolegend | 101218 |
| CD11b (M1/70) | PE | BD | 553311 |
| Ly6G (1A8) | BV421 | BD | 562737 |
| I-A/I-E MHC-II (M5/114.15.2) | PECy7 | eBioscience | 25-5321-82 |
| I-A/I-E MHC-II (M5/114.15.2) | BV711 | Biolegend | 107643 |
| PD-L1/CD274 (MIH5) | BV711 | BD | 563369 |
| CD31 (390) | PECy7 | Biolegend | 102418 |
| PDPN (PMab-1) | A647 | Biolegend | 156204 |
| CD140a (APA5) | BV605 | Biolegend | 135916 |
| CD206 (C068C2) | A647 | Biolegend | 141712 |
| α-SMA (1A4) | Cy3 | Sigma-Aldrich | C6198 |
| Rat IgG2a, λ Isotype Control (B39-4) | BV711 | BD | 563394 |
| Rat IgG2b, κ Isotype Control (RTK4530) | BV711 | Biolegend | 400653 |
| Rat IgG2a, κ Isotype Control (RTK2758) | A647 | Biolegend | 400526 |

**Table S4**. IC_50_ values for each cell line with respective standard deviation (SD).

| Cell Line | IC_50_±SD/µM |
| --- | --- |
| 253J | 3.7±0.7 |
| 5637 | 0.33±0.08 |
| J82 | 4.8±0.3 |
| RT112 | 3.3±0.2 |
| TCCSUP | 2.4±0.3 |
| 4K5 | 3.1±0.2 |

**Table S5**. Mean concentration (pg/mL) with respective standard deviation and fold-change with respective standard error of the mean value of each cytokine in each treatment group. GM-CSF, IL-10 and IFN-α did not show any differences compared to the control group and were not represented in Figure 5i.

|  | Treatment groups | Control | Anti-PD-L1 | CM-1758 | CM-1758  +Anti-PD-L1 |
| --- | --- | --- | --- | --- | --- |
| IFN-γ | Concentration (pg/mL) | 9.1±0.8 | 13±3 | 12±4 | 15±2 |
|  | Fold-change | 1 | 1.4±0.1 | 1.3±0.2 | 1.69±0.08 |
| CXCL1 | Concentration (pg/mL) | 87±44 | 120±65 | 86±54 | 92±45 |
|  | Fold-change | 1 | 1.4±0.3 | 1.0±0.3 | 1.0±0.2 |
| TNF-α | Concentration (pg/mL) | 22±3 | 28±5 | 26±2 | 26±7 |
|  | Fold-change | 1 | 1.3±0.1 | 1.20±0.04 | 1.2±0.1 |
| CCL2 | Concentration (pg/mL) | 130±11 | 143±13 | 194±90 | 142±20 |
|  | Fold-change | 1 | 1.09±0.04 | 1.5±0.3 | 1.09±0.07 |
| IL-12 | Concentration (pg/mL) | 11.1±0.5 | 11±1 | 12±1 | 14±4 |
|  | Fold-change | 1 | 1.02±0.04 | 1.03±0.05 | 1.2±0.1 |
| CCL5 | Concentration (pg/mL) | 43±17 | 71±36 | 36±14 | 68±24 |
|  | Fold-change | 1 | 1.6±0.4 | 0.8±0.1 | 1.6±0.2 |
| IL-1β | Concentration (pg/mL) | 44±3 | 48±8 | 43±2 | 46±4 |
|  | Fold-change | 1 | 1.09±0.07 | 0.99±0.02 | 1.05±0.03 |
| CXCL10 | Concentration (pg/mL) | 1392±484 | 2384±814 | 1706±487 | 2146±517 |
|  | Fold-change | 1 | 1.7±0.2 | 1.2±0.1 | 1.5±0.1 |
| GM-CSF | Concentration (pg/mL) | 47±0 | 47±0 | 51±4 | 49±4 |
|  | Fold-change | 1 | 1±0 | 1.07±0.03 | 1.04±0.03 |
| IL-10 | Concentration (pg/mL) | 249±0 | 254±12 | 250±3 | 256±17 |
|  | Fold-change | 1 | 1.02±0.02 | 1.004±0.004 | 1.03±0.03 |
| IFN-β | Concentration (pg/mL) | 3±1 | 4±3 | 3±2 | 2±2 |
|  | Fold-change | 1 | 1.7±0.6 | 1.0±0.3 | 1.0±0.3 |
| IFN-α | Concentration (pg/mL) | 19.9±0.3 | 20±1 | 20.0±0.5 | 20±1 |
|  | Fold-change | 1 | 1.02±0.02 | 1.007±0.009 | 1.03±0.02 |
| IL-6 | Concentration (pg/mL) | 25±4 | 24±2 | 29±7 | 28±8 |
|  | Fold-change | 1 | 0.95±0.03 | 1.1±0.1 | 1.1±0.1 |

**Supplementary Methods**

*DNA extraction and sodium-bisulfite modification*

For DNA extraction, cell pellets were digested with lysis buffer (Tris-HCl 100 mM pH 8.5, EDTA 5 mM, SDS 0.2%, NaCl 200 mM and proteinase K 500 μg/mL) for 2 h at 55ºC with shaking. Then, isopropanol was added and the DNA pellet was washed two times with ethanol 70% and resuspended in TE buffer (Tris-HCl 10 mM pH 8, EDTA 1 mM). DNA concentration was measured on a Qubit 4 Fluorometer (Thermo Fisher Scientific, USA) using the Qubit DNA broad range assay kit (Thermo Fisher Scientific, USA). Fifty ng of DNA were bisulfite-modified using EZ DNA Methylation-Gold™ Kit (Zymo Research, USA), according to the manufacturer’s recommendations. The bisulfite-converted DNA was eluted in 40 µL of sterile distilled water and stored at −80 °C until further use.

*Quantitative methylation-specific PCR (qMSP)*

*PD-L1* promoter methylation levels were evaluated by qMSP using sodium-bisulfite modified DNA. Two CGs of interest in *PD-L1* promoter associated with PD-L1 expression were selected (cg02823866 and cg14305799) using the Shiny Methylation Analysis Resource Tool (SMART). *ACTB* was used as a housekeeping gene and specific primer sequences can be found in Table S1. One µL of modified DNA, 5 µL Xpert Fast SYBR (GRiSP, Portugal) and 0.3 µL of each primer pair were used in the qMSP reaction. Each sample was run in triplicate in 384-well plates in a QuantStudio 12K Flex Real-Time PCR (Thermo Fisher Scientific, USA). A standard curve using the methylated positive control (5x dilution factor) for PCR efficiency evaluation was included, along with a negative non-methylated control. Relative methylation levels were calculated as the ratio between the mean methylation levels of each primers pair and the respective value for *ACTB* for each sample.
